# Component response rate variation underlies the stability of highly complex finite systems

**DOI:** 10.1038/s41598-020-64401-w

**Published:** 2020-05-19

**Authors:** A. Bradley Duthie

**Affiliations:** Biological and Environmental Sciences, University of Stirling, Stirling, FK9 4LA UK

**Keywords:** Biochemical reaction networks, Dynamic networks, Ecological networks

## Abstract

The stability of a complex system generally decreases with increasing system size and interconnectivity, a counterintuitive result of widespread importance across the physical, life, and social sciences. Despite recent interest in the relationship between system properties and stability, the effect of variation in response rate across system components remains unconsidered. Here I vary the component response rates (*γ*) of randomly generated complex systems. I use numerical simulations to show that when component response rates vary, the potential for system stability increases. These results are robust to common network structures, including small-world and scale-free networks, and cascade food webs. Variation in *γ* is especially important for stability in highly complex systems, in which the probability of stability would otherwise be negligible. At such extremes of simulated system complexity, the largest stable complex systems would be unstable if not for variation in *γ*. My results therefore reveal a previously unconsidered aspect of system stability that is likely to be pervasive across all realistic complex systems.

## Introduction

In 1972, May^1^ first demonstrated that randomly assembled systems of sufficient complexity are almost inevitably unstable given infinitesimally small perturbations. Complexity in this case is defined by the size of the system (i.e., the number of potentially interacting components; $S$), its connectance (i.e., the probability that one component will interact with another; $C$), and the variance of interaction strengths (${\sigma }^{2}$)^2^. May’s finding that the probability of local stability falls to near zero given a sufficiently high threshold of $\sigma \sqrt{SC}$ is broadly relevant for understanding the dynamics and persistence of systems such as ecological^1–6^, neurological^7,8^, biochemical^9,10^, and socio-economic^11–14^ networks. As such, identifying general principles that affect stability in complex systems is of wide-ranging importance.

**Figure 1:**
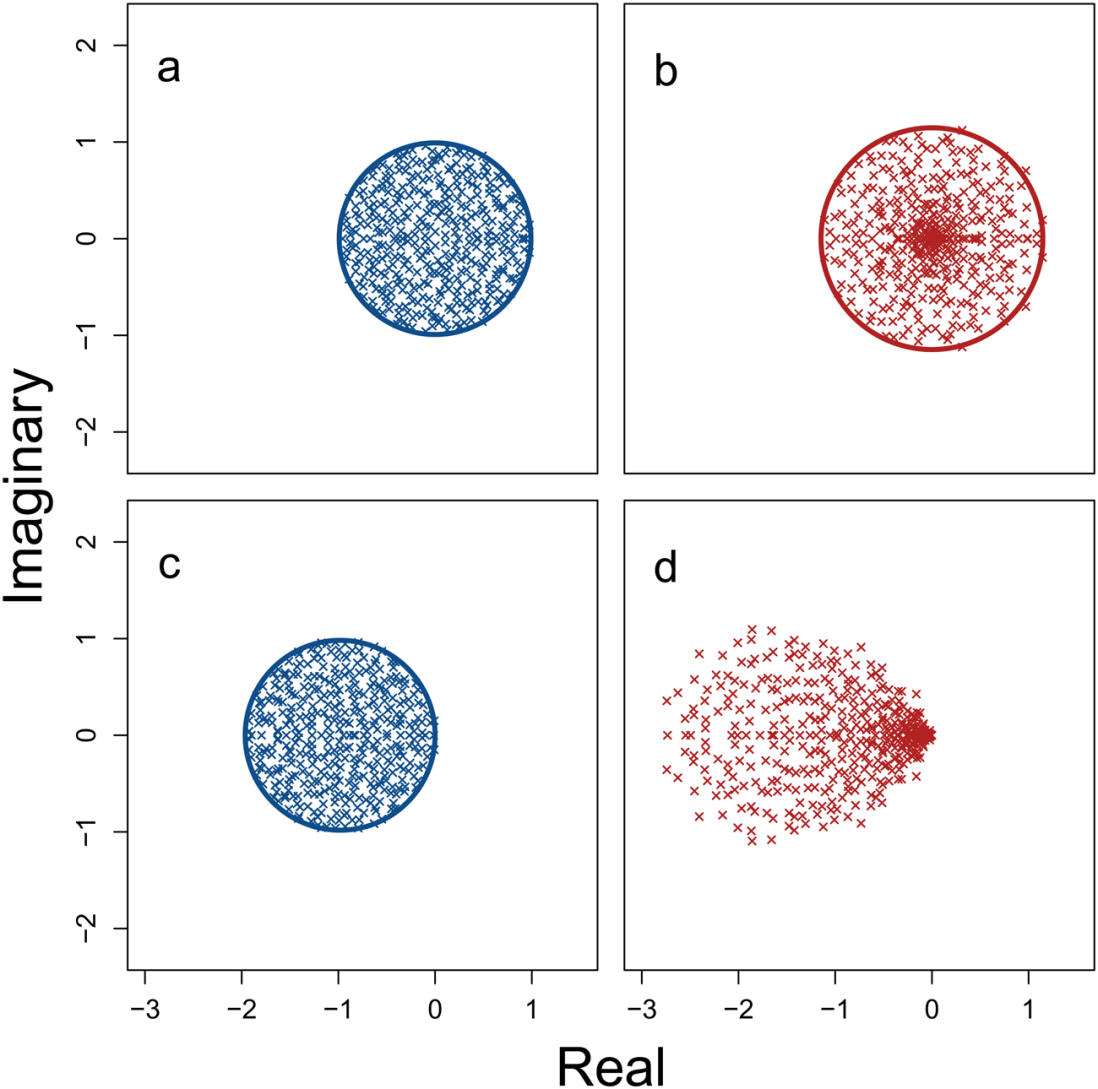
Eigenvalue distributions of random complex systems. Each panel shows the real (x-axis) and imaginary (y-axis) parts of $S=400$ eigenvalues from random $S\times S$ matrices. (**a**) A system represented by a matrix **A**, in which all elements are sampled from a normal distribution with $\mu =0$ and ${\sigma }_{A}=1/\sqrt{S}$. Points are uniformly distributed within the blue circle centred at the origin with a radius of ${\sigma }_{A}\sqrt{S}=1$. (**b**) The same system as in **a** after including variation in the response rates of $S$ components, represented by the diagonal matrix $\gamma$, such that ${\bf{M}}=\gamma {\bf{A}}$. Elements of $\gamma$ are randomly sampled from a uniform distribution from ${\rm{\min }}\,=0$ to ${\rm{\max }}\,=2$. Eigenvalues of ${\bf{M}}$ are then distributed non-uniformly within the red circle centred at the origin with a radius of $\sqrt{{\sigma }_{A}^{2}(1+{\sigma }_{\gamma }^{2})S}\approx 1.15$. (**c**) A different random system ${\bf{A}}$ constructed from the same parameters as in **a**, except with diagonal element values of −1. (**d**) The same system **c** after including variation in component response rates, sampled from ${\mathscr{U}}(0,2)$ as in **b**.

Randomly assembled complex systems can be represented as large square matrices (${\bf{M}}$) with $S$ components (e.g., networks of species^2^ or banks^12^). One element of such a matrix, ${M}_{ij}$, defines how component $j$ affects component $i$ in the system at a point of equilibrium^2^. Off-diagonal elements ($i\ne j$) therefore define interactions between components, while diagonal elements ($i=j$) define component self-regulation (e.g., carrying capacity in ecological communities). Traditionally, off-diagonal elements are assigned non-zero values with a probability $C$, which are sampled from a distribution with variance ${\sigma }^{2}$; diagonal elements are set to −1^1,2,5^. Local system stability is assessed using eigenanalysis on ${\bf{M}}$, with the system being stable if the real parts of all eigenvalues ($\lambda$), and therefore the leading eigenvalue (${\lambda }_{max}$), are negative ($\Re ({\lambda }_{max}) < 0$)^1,2^. In a large system (high $S$), eigenvalues are distributed uniformly^15^ within a circle centred at $\Re =-\,d$ (−*d* is the mean value of diagonal elements) and $\Im =0$, with a radius of $\sigma \sqrt{SC}$^1,2,5^ (Fig. 1a). Local stability of randomly assembled systems therefore becomes increasingly unlikely as $S$, $C$, and $\sigma$ increase.

May’s^1,2^ stability criterion $\sigma \sqrt{SC} < d$ assumes that the expected response rates ($\gamma$) of individual components to perturbations of the system are identical, but this is highly unlikely in any complex system. In ecological communities, for example, the rate at which population density changes following perturbation will depend on the generation time of organisms, which might vary by orders of magnitude among species. Species with short generation times will respond quickly (high $\gamma$) to perturbations relative to species with long generation times (low $\gamma$). Similarly, the speed at which individual banks respond to perturbations in financial networks, or individuals or institutions respond to perturbations in complex social networks, is likely to vary. The effect of such variance on stability has not been investigated in complex systems theory. Intuitively, variation in $\gamma$ (${\sigma }_{\gamma }^{2}$) might be expected to decrease system stability by introducing a new source of variation into the system and thereby increasing $\sigma$. Here I show that, despite higher $\sigma$, realistic complex systems (in which $S$ is high but finite) are actually more likely to be stable if their individual component response rates vary. My results are robust across commonly observed network structures, including random^1^, small-world^16^, scale-free^17^, and cascade food web^18,19^ networks.

## Results

### Component response rates of random complex systems

Complex systems (${\bf{M}}$) are built from two matrices, one modelling component interactions (${\bf{A}}$), and second modelling component response rates (***γ***). Both ${\bf{A}}$ and ***γ*** are square $S\times S$ matrices. Rows in ${\bf{A}}$ define how a given component $i$ is affected by each component $j$ in the system, including itself (where $i=j$). Off-diagonal elements of ${\bf{A}}$ are independent and identically distributed (i.i.d.), and diagonal elements are set to ${A}_{ii}=-\,1$ as in May^1^. Diagonal elements of ***γ*** are positive, and off-diagonal elements are set to zero (i.e., ${\boldsymbol{\gamma }}$ is a diagonal matrix with positive support). The distribution of *diag*(***γ***) over $S$ components thereby models the distribution of component response rates. The dynamics of the entire system ${\bf{M}}$ can be defined as follows^20^,

1 $${\bf{M}}={\boldsymbol{\gamma }}{\bf{A}}.$$

Equation 1 thereby serves as a null model to investigate how variation in component response rate (${\sigma }_{\gamma }^{2}$) affects complex systems. In the absence of such variation (${\sigma }_{\gamma }^{2}=0$), ***γ*** is set to the identity matrix (diagonal elements all equal 1) and ${\bf{M}}={\bf{A}}$. Under these conditions, eigenvalues of ${\bf{M}}$ are distributed uniformly^15^ in a circle centred at $(\,-\,1,0)$ with a radius of $\sigma \sqrt{SC}$^1^ (Fig. 1a).

### Effect of ${\sigma }_{\gamma }^{2}$ on M (co)variation

The value of $\Re ({\lambda }_{max})$, and therefore system stability, can be estimated from five properties of ${\bf{M}}$^21^. These properties include (1) system size ($S$), (2) mean self-regulation of components ($d$), (3) mean interaction strength between components ($\mu$), (4) the variance of between component interaction strengths (hereafter ${\sigma }_{M}^{2}$, to distinguish from ${\sigma }_{A}^{2}$ and ${\sigma }_{\gamma }^{2}$), and (5) the correlation of interaction strengths between components, ${M}_{ij}$ and ${M}_{ji}$ ($\rho$)^22^. Positive ${\sigma }_{\gamma }^{2}$ does not change $S$, nor does it necessarily change $E[d]$ or $E[\mu ]$. What ${\sigma }_{\gamma }^{2}$ does change is the total variation in component interaction strengths (${\sigma }_{M}^{2}$), and $\rho$. Introducing variation in $\gamma$ increases the total variation in the system. Variation in the off-diagonal elements of ${\bf{M}}$ is described by the joint variation of two random variables,

2 $${\sigma }_{M}^{2}={\sigma }_{A}^{2}{\sigma }_{\gamma }^{2}+{\sigma }_{A}^{2}E{[{\gamma }_{i}]}^{2}+{\sigma }_{\gamma }^{2}E{[{A}_{ij}]}^{2}.$$

Given $E[{\gamma }_{i}]=1$ and $E[{A}_{ij}]=0$, Eq. 2 can be simplified,

$${\sigma }_{M}^{2}={\sigma }_{A}^{2}(1+{\sigma }_{\gamma }^{2}).$$

The increase in ${\sigma }_{M}^{2}$ caused by ${\sigma }_{\gamma }^{2}$ can be visualised from the eigenvalue spectra of **A** versus ${\bf{M}}=\gamma {\bf{A}}$ (Fig. 1). Given $d=0$ and $C=1$, the distribution of eigenvalues of **A** and **M** lie within a circle of a radius ${\sigma }_{A}\sqrt{S}$ and ${\sigma }_{M}\sqrt{S}$, respectively (Fig. 1a vs. 1b). If $d\ne 0$, positive ${\sigma }_{\gamma }^{2}$ changes the distribution of eigenvalues^23–25^, potentially affecting stability (Fig. 1c vs. 1d).

Given ${\sigma }_{\gamma }^{2}=0$, $\Re ({\lambda }_{max})$ increases linearly with $\rho$ such that^26^,

$$\Re ({\lambda }_{max})\approx {\sigma }_{M}\sqrt{SC}(1+\rho )\mathrm{}.$$

**Figure 2:**
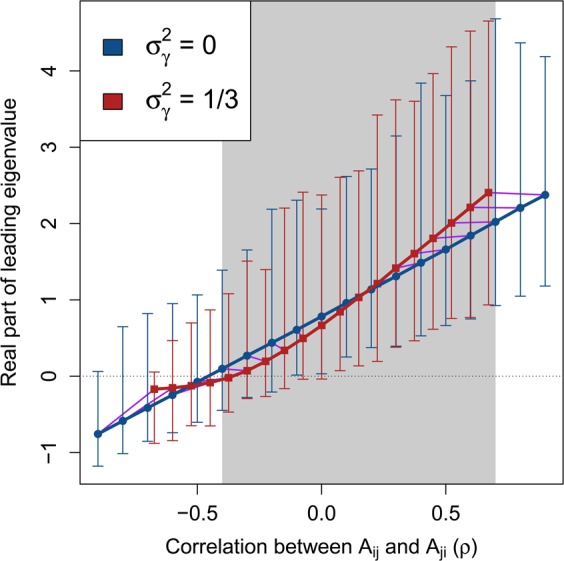
Complex system correlation versus stability with and without variation in component response rates. Each point represents 10000 replicate numerical simulations of a random complex system ${\bf{M}}=\gamma {\bf{A}}$ with a fixed correlation between off-diagonal elements ${A}_{ij}$ and ${A}_{ji}$ ($\rho$, x-axis). Where real parts of eigenvalues of ${\bf{M}}$ are negative (y-axis), ${\bf{M}}$ is stable (black dotted line). Blue circles show systems in the absence of variation in component response rates (${\sigma }_{\gamma }^{2}=0$). Red squares show systems in which ${\sigma }_{\gamma }^{2}=1/3$. Arrows show the range of real parts of leading eigenvalues observed. Because $\gamma$ decreases the magnitude of $\rho$, purple lines are included to link replicate simulations before (blue circles) and after (red squares) including $\gamma$. The range of $\rho$ values in which $\gamma$ decreases the mean real part of the leading eigenvalue is indicated with grey shading. In all simulations, system size and connectance were set to $S=25$ and $C=1$, respectively. Off-diagonal elements of **A** were randomly sampled from ${A}_{ij}\sim {\mathscr{N}}{\mathrm{(0,0.4}}^{2})$, and diagonal elements were set to −1. Elements of $\gamma$ were sampled, $\gamma \sim {\mathscr{U}}\mathrm{(0,2)}$.

If $\rho  < 0$, such as when ${\bf{M}}$ models a predator-prey system in which ${M}_{ij}$ and ${M}_{ji}$ have opposing signs, stability increases^2^. If diagonal elements of ***γ*** vary independently, the magnitude of $\rho$ is decreased because ${\sigma }_{\gamma }^{2}$ increases the variance of ${M}_{ij}$ without affecting the expected covariance between ${M}_{ij}$ and ${M}_{ji}$ (Fig. 2).

### Numerical simulations of random systems with and without ${\sigma }_{\gamma }^{2}$

I used numerical simulations and eigenanalysis to test how variation in $\gamma$ affects stability in random matrices with known properties, comparing the stability of **A** versus ${\bf{M}}=\gamma {\bf{A}}$. Values of $\gamma$ were sampled from a uniform distribution where $\gamma \sim {\mathcal{U}}(0,2)$ and ${\sigma }_{\gamma }^{2}=1/3$ (see Supplementary Information for other $\gamma$ distributions, which gave similar results). In all simulations, diagonal elements were standardised to ensure that −*d* between individual $A$ and $m$ pairs were identical (also note that $E[{\gamma }_{i}]=1$). First I focus on the effect of $\gamma$ across values of $\rho$, then for increasing system sizes ($S$) in random and structured networks. By increasing $S$, the objective is to determine the effect of $\gamma$ as system complexity increases toward the boundary at which stability is realistic for a finite system.

### Simulation of random M across *ρ*

Numerical simulations revealed that ${\sigma }_{\gamma }^{2}$ results in a nonlinear relationship between $\rho$ and $\Re ({\lambda }_{max})$, which can sometimes increase the stability of the system. Figure 2 shows a comparison of $\Re ({\lambda }_{max})$ across $\rho$ values for ${\bf{A}}$ (${\sigma }_{\gamma }^{2}=0$) versus **M** (${\sigma }_{\gamma }^{2}=1/3$) given $S=25$, $C=1$, and ${\sigma }_{A}=0.4$. For $-\,0.4\le \rho \le 0.7$ (shaded region of Fig. 2), expected $\Re ({\lambda }_{max})$ was lower in ${\bf{M}}$ than ${\bf{A}}$. For $\rho \ge -\,0.1$, the lower bound of the range of $\Re ({\lambda }_{max})$ values also decreased given ${\sigma }_{\gamma }^{2}$, resulting in negative $\Re ({\lambda }_{max})$ in ${\bf{M}}$ for $\rho =-\,0.1$ and $\rho =0$. Hence, across a wide range of system correlations, variation in the response rate of system components had a stabilising effect.

**Figure 3:**
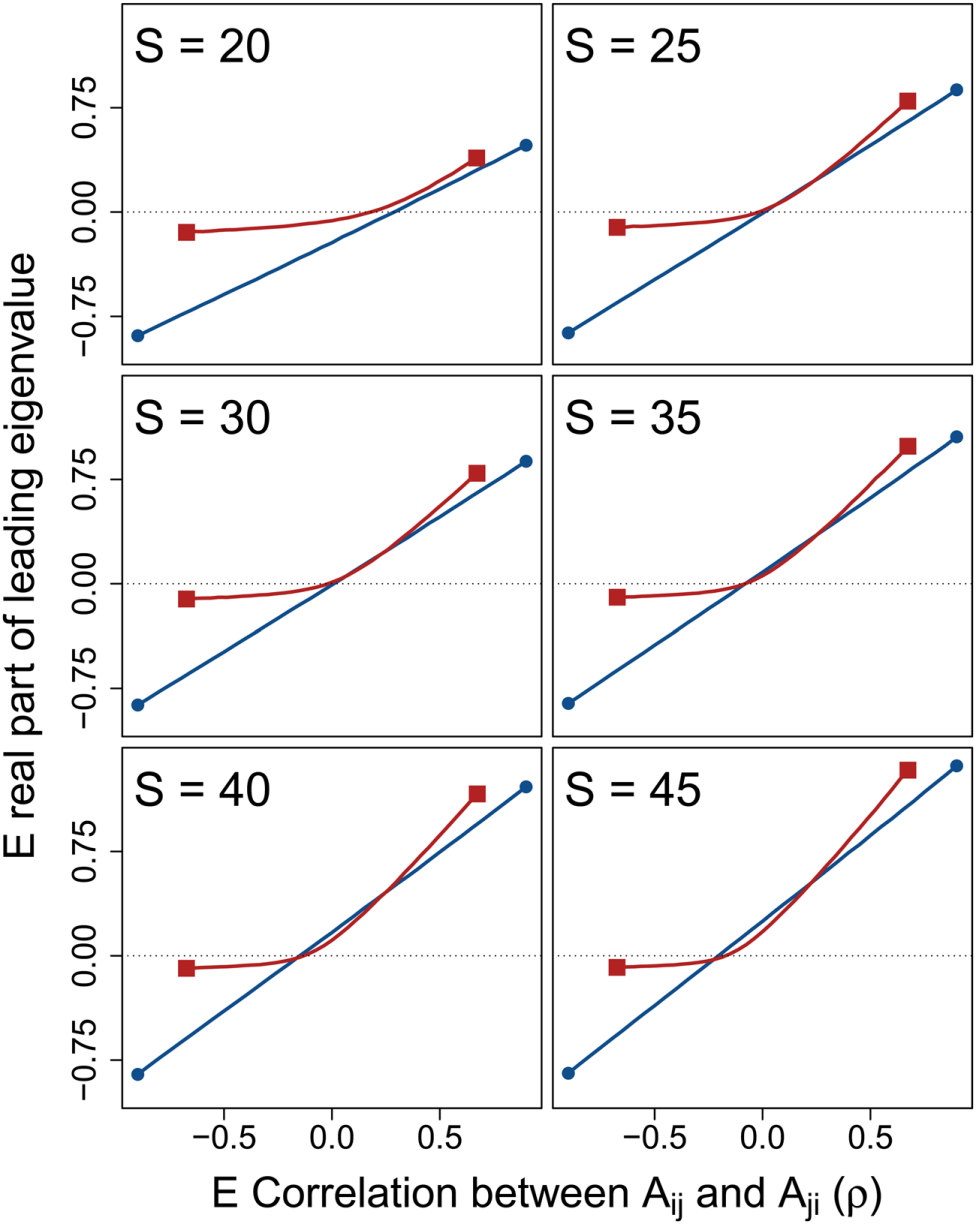
System correlation versus stability across different system sizes. In each panel, 10000 random complex systems ${\bf{M}}=\gamma {\bf{A}}$ are simulated for each correlation $\rho =\{\,-\,0.90,-\,0.85,\ldots ,0.85,0.90\}$ between off-diagonal elements ${A}_{ij}$ and ${A}_{ji}$. Lines show the expected real part of the leading eigenvalues of **M** (red squares; ${\sigma }_{\gamma }^{2}=1/3$) versus **A** (blue circles; ${\sigma }_{\gamma }^{2}=0$) across $\rho$, where negative values (below the dotted black line) indicate system stability. Differences between lines thereby show the effect of component response rate variation ($\gamma$) on system stability across system correlations and sizes ($S$). For all simulations, system connectance was $C=1$. Off-diagonal elements of **A** were randomly sampled from ${A}_{ij}\sim {\mathscr{N}}(0,{0.2}^{2})$, and diagonal elements were set to −1. Elements of $\gamma$ were sampled such that $\gamma \sim {\mathscr{U}}(0,2)$, so ${\sigma }_{\gamma }^{2}=1/3$.

The stabilising effect of ${\sigma }_{\gamma }^{2}$ across $\rho$ increased with increasing $S$. Figure 3 shows numerical simulations of ${\bf{M}}$ across increasing $S$ given $C=1$ and ${\sigma }_{A}=0.2$ (${\sigma }_{A}$ has been lowered here to better illustrate the effect of $S$; note that now given $S=25$, $1={\sigma }_{A}\sqrt{SC}$). For relatively small systems ($S\le 25$), ${\sigma }_{\gamma }^{2}$ never decreased the expected $\Re ({\lambda }_{max})$. But as $S$ increased, the curvilinear relationship between $\rho$ and $\Re ({\lambda }_{max})$ decreased expected $\Re ({\lambda }_{max})$ for ${\bf{M}}$ given low magnitudes of $\rho$. In turn, as $S$ increased, and systems became more complex, ${\sigma }_{\gamma }^{2}$ increased the proportion of numerical simulations that were observed to be stable (see below).

### Simulation of random M across S

**Figure 4:**
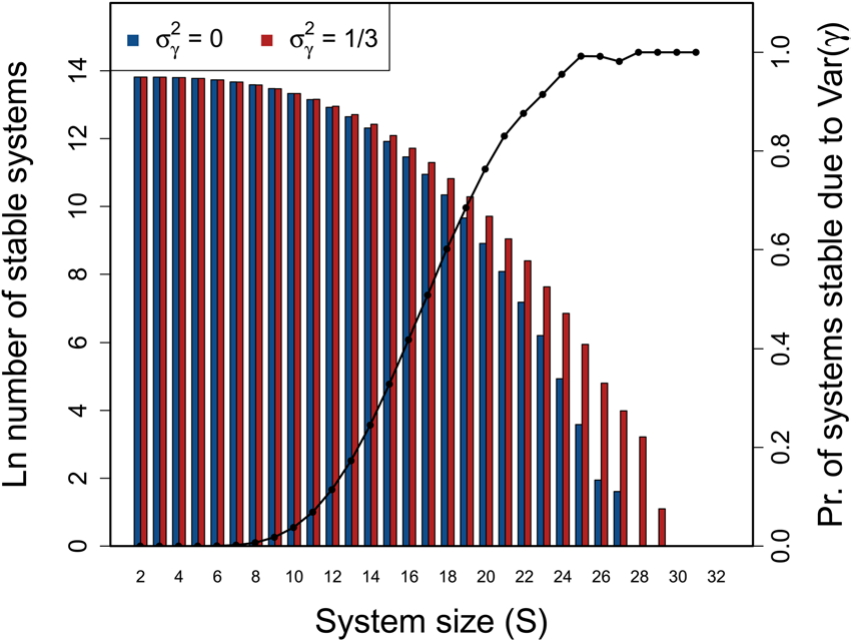
Stability of large complex systems with and without variation in component response rate (*γ*). The log number of systems that are stable across different system sizes ($S=\{2,3,\ldots ,49,50\}$) given $C=1$, and the proportion of systems for which variation in $\gamma$ is critical for system stability. For each $S$, 1 million complex systems are randomly generated. Stability of each complex system is tested given variation in $\gamma$ by randomly sampling $\gamma \sim {\mathscr{U}}(0,2)$. Stability given ${\sigma }_{\gamma }^{2} > 0$ is then compared to stability in an otherwise identical system in which ${\gamma }_{i}=E[{\mathscr{U}}(0,2)]$ for all components. Blue and red bars show the number of stable systems in the absence and presence of ${\sigma }_{\gamma }^{2}$, respectively. The black line shows the proportion of systems that are stable when ${\sigma }_{\gamma }^{2} > 0$, but would be unstable if ${\sigma }_{\gamma }^{2}=0$ (i.e., the conditional probability that **A** is unstable given that **M** is stable).

To investigate the effect of ${\sigma }_{\gamma }^{2}$ on stability across systems of increasing complexity, I simulated random ${\bf{M}}=\gamma {\bf{A}}$ matrices at ${\sigma }_{A}=0.4$ and $C=1$ across $S=\{2,3,\ldots ,49,50\}$. One million ${\bf{M}}$ were simulated for each $S$, and the stability of ${\bf{A}}$ vesus ${\bf{M}}$ was assessed given $\gamma \sim {\mathcal{U}}(0,2)$ (${\sigma }_{\gamma }^{2}=1/3$). For all $S > 10$, I found that the number of stable random systems was higher in ${\bf{M}}$ than ${\bf{A}}$ (Fig. 4; see Supplementary Information for full table of results), and that the difference between the probabilities of observing a stable system increased with an increase in $S$. In other words, the potential for ${\sigma }_{\gamma }^{2}$ to affect stability increased with increasing system complexity and was most relevant for systems on the cusp of being too complex to be realistically stable. For the highest values of $S$, nearly all systems that were stable given varying $\gamma$ would not have been stable given $\gamma =1$.

I also simulated 100000 ${\bf{M}}$ for three types of random networks that are typically interpreted as modelling three types of interspecific ecological interactions^2,27^. These interaction types are competitive, mutualist, and predator-prey, as modelled by off-diagonal elements that are constrained to be negative, positive, or paired such that if ${A}_{ij} > 0$ then ${A}_{ji} < 0$, respectively^2^ (but are otherwise identical to the purely random ${\bf{A}}$). As $S$ increased, a higher number of stable ${\bf{M}}$ relative to ${\bf{A}}$ was observed for competitor and predator-prey, but not mutualist, systems. A higher number of stable systems was observed whenever $S > 12$ and $S > 40$ for competitive and predator-prey systems, respectively (note that $\rho  < 0$ for predator-prey systems, making stability more likely overall). The stability of mutualist systems was never affected by ${\sigma }_{\gamma }^{2}$.

The effect of ${\sigma }_{\gamma }^{2}$ on stability did not change qualitatively across values of $C$, ${\sigma }_{A}$, or for different distributions of $\gamma$ (see Supporting Information).

### Simulation of structured M across S

To investigate how ${\sigma }_{\gamma }^{2}$ affects the stability of commonly observed network structures, I simulated one million ${\bf{M}}=\gamma {\bf{A}}$ for small-world^16^, scale-free^17^, and cascade food web^18,19^ networks. In all of these networks, rules determining the presence or absence of an interaction between components $i$ and $j$ constrain the overall structure of the network. In small-world networks, interactions between components are constrained so that the expected degree of separation between any two components increases in proportion to ${\rm{l}}{\rm{o}}{\rm{g}}(S)$^16^. In scale-free networks, the distribution of the number of components with which a focal component interacts follows a power law; a few components have many interactions while most components have few interactions^17^. In cascade food webs, species are ranked and interactions are constrained such that a species $i$ can only feed on $j$ if the rank of $i > j$.

Network structure did not strongly modulate the effect that ${\sigma }_{\gamma }^{2}$ had on stability. For comparable magnitudes of complexity, structured networks still had a higher number of stable ${\bf{M}}$ than ${\bf{A}}$. For random networks, ${\sigma }_{\gamma }^{2}$ increased stability given $S > 10$ (${\sigma }_{A}=0.4$ and $C=1$), and therefore complexity ${\sigma }_{A}\sqrt{SC}\,\gtrapprox \,1.26$. This threshold of complexity, above which more ${\bf{M}}$ than ${\bf{A}}$ were stable, was comparable for small-world networks, and slightly lower for scale-free networks (note that algorithms for generating small-world and scale-free networks necessarily led to varying $C$; see methods). Varying $\gamma$ increased stability in cascade food webs for $S > 27$, and therefore at a relatively low complexity magnitudes compared to random predator-prey networks ($S > 40$). Overall, network structure did not greatly change the effect that ${\sigma }_{\gamma }^{2}$ had on increasing the upper bound of complexity within which stability might reasonably be observed.

### System feasibility given ${\sigma }_{\gamma }^{2}$

For complex systems in which individual system components represent the density of some tangible quantity, it is relevant to consider the feasibility of the system. Feasibility assumes that values of all components are positive at equilibrium^6,28,29^. This is of particular interest for ecological communities because population density ($n$) cannot take negative values, meaning that ecological systems need to be feasible for stability to be biologically realistic^28^. While my results are intended to be general to all complex systems, and not restricted to species networks, I have also performed a feasibility analysis on all matrices tested for stability. I emphasise that $\gamma$ is not interpreted as population density in this analysis, but instead as a fundamental property of species life history such as expected generation time. Feasibility was unaffected by ${\sigma }_{\gamma }^{2}$ and instead occurred with a fixed probability of ${\mathrm{1/2}}^{S}$, consistent with a recent proof by Serván *et al*.^30^ (see Supplementary Information). Hence, for pure interacting species networks, variation in component response rate (i.e., species generation time) does not affect stability at biologically realistic species densities.

### Targeted manipulation of *γ*

To further investigate the potential of ${\sigma }_{\gamma }^{2}$ to be stabilising, I used a genetic algorithm. Genetic algorithms are heuristic tools that mimic evolution by natural selection, and are useful when the space of potential solutions (in this case, possible combinations of $\gamma$ values leading to stability in a complex system) is too large to search exhaustively^31^. Generations of selection on $\gamma$ value combinations to minimise $\Re ({\lambda }_{max})$ demonstrated the potential for ${\sigma }_{\gamma }^{2}$ to increase system stability. Across $S=\{2,3,\ldots ,39,40\}$, sets of $\gamma$ values were found that resulted in stable systems with probabilities that were up to four orders of magnitude higher than when $\gamma =1$ (see Supplementary Information), meaning that stability could often be achieved by manipulating $S$ $\gamma$ values rather than $S\times S$ ${\bf{M}}$ elements (i.e., by manipulating component response rates rather than interactions between components).

## Discussion

I have shown that the stability of complex systems might often be contigent upon variation in the response rates of their individual components, meaning that factors such as rate of trait evolution (in biological networks), transaction speed (in economic networks), or communication speed (in social networks) need to be considered when investigating the stability of complex systems. Variation in component response rate is more likely to be critical for stability in systems that are especially complex, and it can ultimately increase the probability that system stability is observed above that predicted by May’s^1^ classically derived $\sigma \sqrt{SC}$ criterion. The logic outlined here is general, and potentially applies to any complex system in which individual system components can vary in their reaction rates to system perturbation.

It is important to recognise that variation in component response rate is not stabilising per se; that is, adding variation in component response rates to a particular system does not increase the probability that the system will be stable. Rather, highly complex systems that are observed to be stable are more likely to have varying component response rates, and for this variation to be critical to their stability (Fig. 4). This is caused by the shift to a non-uniform distribution of eigenvalues that occurs by introducing variation in $\gamma$ (Fig. 1), which can sometimes cause all of the real components of the eigenvalues of the system matrix to become negative, but might also increase the real components of eigenvalues.

My focus here is distinct from Gibbs *et al*.^24^, who applied the same mathematical framework to investigate how a diagonal matrix ${\bf{X}}$ (equivalent to ${\boldsymbol{\gamma }}$ in my model) affects the stability of a community matrix ${\bf{M}}$ given an interaction matrix ${\bf{A}}$ within a generalised Lotka-Volterra model, where ${\bf{M}}={\bf{X}}{\bf{A}}$. Gibbs *et al*.^24^ analytically demonstrated that the effect of ${\bf{X}}$ on system stability decreases exponentially as system size becomes arbitrarily large ($S\to \infty$) for a given magnitude of complexity $\sigma \sqrt{SC}$. My numerical results do not contradict this prediction because I did not scale $\sigma =1/\sqrt{S}$, but instead fixed $\sigma$ and increased $S$ to thereby increase total system complexity (see Supplemental Information for results simulated across $\sigma$ and $C$). Overall, I show that component response rate variation increases the upper bound of complexity at which stability can be realistically observed, meaning that highly complex systems are more likely than not to vary in their component response rates, and for this variation to be critical for system stability.

Interestingly, while complex systems were more likely to be stable given variation in component response rate, they were not more likely to be feasible, meaning that stability was not increased when component values were also restricted to being positive at equilibrium. Feasibility is important to consider, particularly for the study of ecological networks of species^6,25,28,30^ because population densities cannot realistically be negative. My results therefore suggest that variation in the rate of population responses to perturbation (e.g., due to differences in generation time among species) is unlikely to be critical to the stability of purely multi-species interaction networks (see also Supplementary Information). Nevertheless, ecological interactions do not exist in isolation in empirical systems^20^, but instead interact with evolutionary, abiotic, or social-economic systems. The relevance of component response rate for complex system stability should therefore not be ignored in the broader context of ecological communities.

The potential importance of component response rate variation was most evident from the results of simulations in which the genetic algorithm was used in attempt to maximise the probability of system stability. The probability that some combination of component response rates could be found to stabilise the system was shown to be up to four orders of magnitude higher than the background probabilities of stability in the absence of any component response rate variation. Instead of manipulating the $S\times S$ interactions between system components, it might therefore be possible to manipulate only the $S$ response rates of individual system components to achieve stability. Hence, managing the response rates of system components in a targeted way could potentially facilitate the stabilisation of complex systems through a reduction in dimensionality.

A general mathematical framework encompassing shifts in eigenvalue distributions caused by a diagonal matrix *γ* has been investigated^23^ and recently applied to questions concerning species density and feasibility^24,25^, but *γ* has not been interpreted as rates of response of individual system components to perturbation. My model focuses on component response rates for systems of a finite size, in which complexity is high but not yet high enough to make the probability of stability unrealistically low for actual empirical systems. For this upper range of system size, randomly assembled complex systems are more likely to be stable if their component response rates vary (e.g., $10 < S < 30$ for parameter values in Fig. 4). Variation in component response rate might therefore be critical for maintaining stability in many highly complex empirical systems. These results are broadly applicable for understanding the stability of complex networks across the physical, life, and social sciences.

## Methods

### Component response rate (*γ*) variation

In a synthesis of eco-evolutionary feedbacks on community stability, Patel *et al*.^20^ model a system that includes a vector of potentially changing species densities (**n**) and a vector of potentially evolving traits (**x**). For any species $i$ or trait $j$, change in species density (${n}_{i}$) or trait value (${x}_{j}$) with time ($t$) is a function of the vectors **n** and **x**,

$$\frac{d{n}_{i}}{dt}={n}_{i}{f}_{i}({\bf{n}},{\bf{x}}),$$

$$\frac{d{x}_{j}}{dt}=\epsilon {g}_{j}({\bf{n}},{\bf{x}}\mathrm{)}.$$

In the above, ${f}_{i}$ and ${g}_{j}$ are functions that define the effects of all species densities and trait values on the density of a species $i$ and the value of trait $j$, respectively. Patel *et al*.^20^ were interested in stability when the evolution of traits was relatively slow or fast in comparison with the change in species densities, and this is modulated in the above by the scalar $\epsilon$. The value of $\epsilon$ thereby determines the timescale separation between ecology and evolution, with high $\epsilon$ modelling relatively fast evolution and low $\epsilon$ modelling relatively slow evolution^20^.

I use the same principle that Patel *et al*.^20^ use to modulate the relative rate of evolution to modulate rates of component responses for $S$ components. Following May^1,32^, the value of a component $i$ at time $t$ (${v}_{i}(t)$) is affected by the value of $j$ (${v}_{j}(t)$) and $j$’s marginal effect on $i$ (${a}_{ij}$), and by $i$’s response rate (${\gamma }_{i}$),

$$\frac{d{v}_{i}(t)}{dt}={\gamma }_{i}\,\mathop{\sum }\limits_{j=1}^{S}\,{a}_{ij}{v}_{j}(t).$$

In matrix notation^32^,

$$\frac{d{\bf{v}}(t)}{dt}=\gamma {\bf{A}}{\bf{v}}(t)\mathrm{}.$$

In the above, ***γ*** is a diagonal matrix in which elements correspond to individual component response rates. Therefore, ${\bf{M}}={\boldsymbol{\gamma }}{\bf{A}}$ defines the change in values of system components and can be analysed using the techniques of May^1,23,32^. In these analyses, row means of ${\bf{A}}$ are expected to be identical, but variation around this expectation will naturally arise due to random sampling of ${\bf{A}}$ off-diagonal elements and finite $S$. In simulations, the total variation in ${\bf{M}}$ row means that is attributable to ${\bf{A}}$ is small relative to that attributable to ***γ***, especially at high $S$. Variation in ***γ*** specifically isolates the effects of differing component response rates, hence causing differences in expected ${\bf{M}}$ row means.

### Construction of random and structured networks

I used the R programming language for all numerical simulations and analyses^33^. Purely random networks were generated by sampling off-diagonal elements from an i.i.d. ${A}_{ij}\sim {\mathscr{N}}(0,{0.4}^{2})$ with a probability $C$ (unsampled elements were set to zero). Diagonal elements ${A}_{ii}$ were set to −1. Elements of ***γ*** were simulated i.i.d. from a distribution with positive support (typically $\gamma \sim {\mathcal{U}}(0,2)$). Random ${\bf{A}}$ matrices with correlated elements ${A}_{ij}$ and ${A}_{ji}$ were built using Cholesky decomposition. Competitor networks in which off-diagonal elements ${A}_{ij}\le 0$ were constructed by first building a random ${\bf{A}}$, then flipping the sign of any elements in which ${A}_{ij} > 0$. Similarly, mutualist networks were constructed by building a random ${\bf{A}}$, then flipping the sign of elements where ${A}_{ij} < 0$. Predator-prey networks were constructed by first building a random ${\bf{A}}$, then flipping the sign of either ${A}_{ij}$ or ${A}_{ji}$ if ${A}_{ij}\times {A}_{ji} > 0$.

Small-world networks were constructed using the method of Watts and Strogatz^16^. First, a regular network^16^ was created such that components were arranged in a circle. Each component was initially set to interact with its $k\mathrm{/2}$ closest neighbouring components on each side, where $k$ was an even natural number (e.g., for $k=2$ the regular network forms a ring in which each component interacts with its two adjacent neighbours; see Supplemental Material for examples). Each interaction between a focal component and its neighbour was then removed and replaced with with a probability of $\beta$. In replacement, a new component was randomly selected to interact with the focal component; selection was done with equal probability among all but the focal component. The resulting small-world network was represented by a square $S\times S$ binary matrix ${\bf{B}}$ in which 1s represented interactions between components and 0s represented the absence of an interaction. A new random matrix ${\bf{J}}$ was then generated with elements ${J}_{ij}$ sampled i.i.d. from ${\mathscr{N}}(0,{0.4}^{2})$. To build the interaction matrix ${\bf{A}}$, I used element-wise multiplication ${\bf{A}}={\bf{J}}\odot {\bf{B}}$, then set $diag({\bf{A}})=-\,1$. I set $k=S/12$ and simulated small-world networks across all combinations of $S=\mathrm{\{24,}\,\mathrm{48,}\,\mathrm{72,}\,\mathrm{96,}\,\mathrm{120,}\,\mathrm{144,}\,\mathrm{168\}}$ and $\beta =\{0,0.01,0.1,0.25,1\}$.

Scale-free networks were constructed using the method of Albert and Barabási^17^. First, a saturated network (all components interact with each other) of size $m\le S$ was created. New components were then added sequentially to the network; each newly added component was set to interact with $m$ randomly selected existing components. When the system size reached $S$, the distribution of the number of total interactions that components had followed a power-law tail^17^. The resulting network was represented by an $S\times S$ binary matrix ${\bf{G}}$, where 1s and 0s represent the presence and absence of an interaction, respectively. As with small-world networks, a random matrix ${\bf{J}}$ was generated, and ${\bf{A}}={\bf{J}}\odot {\bf{G}}$. Diagonal elements were set to −1. I simulated scale-free networks across all combinations of $S=\{24,48,72,96,120\}$ and $m=\{2,3,\ldots ,11,12\}$.

Cascade food webs were constructed following Solow and Beet^18^. First, a random matrix ${\bf{A}}$ was generated with off-diagonal elements sampled i.i.d. so that ${A}_{ij}\sim {\mathscr{N}}{\mathrm{(0,0.4}}^{2})$. Each component in the system was ranked from $1$ to $S$. If component $i$ had a higher rank than component $j$ and ${A}_{ij} < 0$, then ${A}_{ij}$ was multiplied by −1. If $i$ had a lower rank than $j$ and ${A}_{ji} < 0$, then ${A}_{ji}$ was multiplied by −1. In practice, this resulted in a matrix ${\bf{A}}$ with negative and positive values in the lower and upper triangles, respectively. Diagonal elements of ${\bf{A}}$ were set to −1 and $C=1$. I simulated cascade food webs for $S=\{2,3,\ldots ,59,60\}$.

### System feasibility

Dougoud *et al*.^28^ identify the following feasibility criteria for ecological systems characterised by $S$ interacting species with varying densities in a generalised Lotka-Volterra model,

$${{\bf{n}}}^{\ast }=-\,{(\theta {\bf{I}}+{(CS)}^{-\delta }{\bf{J}})}^{-1}{\bf{r}}.$$

In the above, n* is the vector of species densities at equilibrium. Feasibility is satisfied if all elements in n* are positive. The matrix ${\bf{I}}$ is the identity matrix, and the value $\theta$ is the strength of intraspecific competition (diagonal elements). Diagonal values are set to −1, so $\theta =-\,1$. The variable $\delta$ is a normalisation parameter that modulates the strength of interactions ($\sigma$) for ${\bf{J}}$. Implicitly, here $\delta =0$ underlying strong interactions. Hence, ${(CS)}^{-\delta }=1$, so in the above, a diagonal matrix of −1s ($\theta {\bf{I}}$) is added to ${\bf{J}}$, which has a diagonal of all zeros and an off-diagonal affecting species interactions (i.e., the expression ${(CS)}^{-\delta }$ relates to May’s^1^ stability criterion^28^ by $\frac{\sigma }{{(CS)}^{-\delta }}\sqrt{SC} < 1$, and hence for my purposes ${(CS)}^{-\delta }=1$). Given ${\bf{A}}=\theta {\bf{I}}+{\bf{J}}$, the above criteria is therefore reduced to the below (see also Serván *et al*.^30^),

$${{\bf{n}}}^{\ast }=-\,{{\bf{A}}}^{-{\bf{1}}}{\bf{r}}.$$

To check the feasibility criteria for ${\bf{M}}=\gamma {\bf{A}}$, I therefore evaluated $-{{\bf{M}}}^{-{\bf{1}}}{\bf{r}}$ (${\bf{r}}$ elements were sampled i.i.d. from $r\sim {\mathscr{N}}(0,{0.4}^{2})$). Feasibility is satisfied if all of the elements of the resulting vector are positive.

### Genetic algorithm

Ideally, to investigate the potential of ${\sigma }_{\gamma }^{2}$ for increasing the proportion of stable complex systems, the search space of all possible *diag*(***γ***) vectors would be evaluated for each unique ${\bf{M}}=\gamma {\bf{A}}$. This is technically impossible because ${\gamma }_{i}$ can take any real value between 0–2, but even rounding ${\gamma }_{i}$ to reasonable values would result in a search space too large to practically explore. Under these conditions, genetic algorithms are highly useful tools for finding practical solutions by mimicking the process of biological evolution^31^. In this case, the practical solution is finding vectors of *diag*(***γ***) that decrease the most positive real eigenvalue of ${\bf{M}}$. The genetic algorithm used achieves this by initialising a large population of 1000 different potential *diag*(***γ***) vectors and allowing this population to evolve through a process of mutation, crossover (swaping ${\gamma }_{i}$ values between vectors), selection, and reproduction until either a *diag*(***γ***) vector is found where all $\Re (\lambda ) < 0$ or some “giving up” critiera is met.

For each $S=\{2,3,\ldots ,39,40\}$, the genetic algorithm was run for 100000 random ${\bf{M}}=\gamma {\bf{A}}$ (${\sigma }_{A}=0.4$, $C=1$). The genetic algorithm was initialised with a population of 1000 different *diag*(***γ***) vectors with elements sampled i.i.d. from $\gamma \sim {\mathscr{U}}\mathrm{(0,2)}$. Eigenanalysis was performed on the ${\bf{M}}$ resulting from each ***γ***, and the 20 *diag*(***γ***) vectors resulting in ${\bf{M}}$ with the lowest $\Re ({\lambda }_{max})$ each produced 50 clonal offspring with subsequent random mutation and crossover between the resulting new generation of 1000 *diag*(***γ***) vectors. Mutation of each ${\gamma }_{i}$ in a *diag*(***γ***) vector occurred with a probability of 0.2, resulting in a mutation effect of size ${\mathscr{N}}(0,{0.02}^{2})$ being added to generate the newly mutated ${\gamma }_{i}$ (any ${\gamma }_{i}$ values that mutated below zero were multiplied by −1, and any values that mutated above 2 were set to 2). Crossover occurred between two sets of 100 *diag*(***γ***) vectors paired in each generation; vectors were randomly sampled with replacement among but not within sets. Vector pairs selected for crossover swapped all elements between and including two ${\gamma }_{i}$ randomly selected with replacement (this allowed for reversal of vector element positions during crossover; e.g., $\{{\gamma }_{4},{\gamma }_{5},{\gamma }_{6},{\gamma }_{7}\}\to \{{\gamma }_{7},{\gamma }_{6},{\gamma }_{5},{\gamma }_{4}\}$). The genetic algorithm terminated if a stable ${\bf{M}}$ was found, 20 generations occurred, or if the mean ${\boldsymbol{\gamma }}$ fitness increase between generations was less than 0.01 (where fitness was defined as ${W}_{\gamma }=-\,\Re ({\lambda }_{max})$ for **M**).

## Supplementary information

Supplementary Information.

## Data Availability

All code and data are accessible on GitHub.
