# Supplementary material for: Component response rate variation underlies the stability of highly complex finite systems
Source: Sci Rep. 2020 May 19;10:8296. doi: 10.1038/s41598-020-64401-w (PMC7237446; doi:10.1038/s41598-020-64401-w)
Supplement: Supplementary file 1 — Supplementary Information. [file 41598_2020_64401_MOESM1_ESM.pdf]

Component response rate variation underlies the stability of highly complex finite systems (Supplementary Information)

A. Bradley Duthie (alexander.duthie@stir.ac.uk)

Biological and Environmental Sciences, University of Stirling, Stirling, UK, FK9 4LA

This supplemental information supports the manuscript "Component response rate variation underlies the stability of highly complex finite systems" with additional analyses to support its conclusions. All text, code, and data underlying this manuscript are publicly available on GitHub as part of the RandomMatrixStability R package.

The RandomMatrixStability package includes all functions and tools for recreating the text, this supplemental information, and running all code; additional documentation is also provided for package functions. The RandomMatrixStability package is available on GitHub; to download it, the `devtools` library is needed.

```
install.packages("devtools");
library(devtools);
```

The code below installs the RandomMatrixStability package using devtools.

```
install_github("bradduthie/RandomMatrixStability");
```

Supplemental Information table of contents

- Stability across increasing S
- Stability of random ecological networks
  - Competitor networks
  - Mutualist networks
  - Predator-prey networks
- Sensitivity of connectance (C) values
  - C = 0.3
  - C = 0.5
  - C = 0.7
  - C = 0.9
- Large networks of $C = 0.05$ across S and σ
  - $\sigma = 0.3$
  - $\sigma = 0.4$
  - $\sigma = 0.5$
  - $\sigma = 0.6$
- Sensitivity of distribution of γ
- Stability of structured networks
- Feasibility of complex systems
- Stability given targeted manipulation of γ (genetic algorithm)
- Consistency with Gibbs et al. (2018)
- Reproducing simulation results
- Literature cited

Stability across increasing S

Figure 4 of the main text reports the number of stable random complex systems found over 1 million iterations. The table below shows the results for all simulations of random $\mathbf{M}$ matrices at $\sigma_A = 0.4$ and $C = 1$ given a range of $S = \{2, 3, ..., 49, 50\}$. In this table, the `A` refers to $\mathbf{A}$ matrices where $\gamma = 1$, while `M` refers to $\mathbf{M}$ matrices after σ_γ^2 is added and $\gamma \sim \mathcal{U}(0, 2)$. Each row summarises data for a given S over 1 million randomly simulated $\mathbf{M}$. The column `A_unstable` shows the number of $\mathbf{A}$ matrices that are unstable, and the column `A_stable` shows the number of $\mathbf{A}$ matrices that are stable (these two columns sum to 1 million). Similarly, the column `M_unstable` shows the number of $\mathbf{M}$ matrices that are unstable and `M_stable` shows the number that are stable. The columns `A_stabilised` and `A_destabilised` show how many $\mathbf{M}$ matrices were stabilised or destabilised, respectively, by σ_γ^2.

S	A_unstable	A_stable	M_unstable	M_stable	A_stabilised	A_destabilised
2	293	999707	293	999707	0	0
3	3602	996398	3609	996391	0	7
4	14937	985063	15008	984992	0	71
5	39289	960711	39783	960217	36	530
6	78845	921155	80207	919793	389	1751
7	133764	866236	136904	863096	1679	4819
8	204112	795888	208241	791759	5391	9520
9	288041	711959	291775	708225	12619	16353
10	384024	615976	384931	615069	23153	24060
11	485975	514025	481019	518981	35681	30725
12	590453	409547	577439	422561	48302	35288
13	689643	310357	669440	330560	57194	36991
14	777496	222504	751433	248567	60959	34896
15	850159	149841	821613	178387	58567	30021
16	905057	94943	877481	122519	51255	23679
17	943192	56808	919536	80464	40854	17198
18	969018	30982	949944	50056	30102	11028
19	984301	15699	970703	29297	20065	6467
20	992601	7399	983507	16493	12587	3493
21	996765	3235	991532	8468	7030	1797
22	998693	1307	995567	4433	3884	758
23	999503	497	997941	2059	1883	321
24	999861	139	999059	941	899	97
25	999964	36	999617	383	380	33
26	999993	7	999878	122	121	6
27	999995	5	999946	54	53	4
28	1000000	0	999975	25	25	0
29	1000000	0	999997	3	3	0
30	1000000	0	999999	1	1	0
31	1000000	0	999999	1	1	0
32	1000000	0	1000000	0	0	0
33	1000000	0	1000000	0	0	0
34	1000000	0	1000000	0	0	0
35	1000000	0	1000000	0	0	0
36	1000000	0	1000000	0	0	0
37	1000000	0	1000000	0	0	0
38	1000000	0	1000000	0	0	0
39	1000000	0	1000000	0	0	0
40	1000000	0	1000000	0	0	0
41	1000000	0	1000000	0	0	0

S	A_unstable	A_stable	M_unstable	M_stable	A_stabilised	A_destabilised
42	1000000	0	1000000	0	0	0
43	1000000	0	1000000	0	0	0
44	1000000	0	1000000	0	0	0
45	1000000	0	1000000	0	0	0
46	1000000	0	1000000	0	0	0
47	1000000	0	1000000	0	0	0
48	1000000	0	1000000	0	0	0
49	1000000	0	1000000	0	0	0
50	1000000	0	1000000	0	0	0

Overall, the ratio of stable **A** matrices to stable **M** matrices found is greater than 1 whenever $S > 10$ (compare column 3 to column 5), and this ratio increases with increasing S (column 1). Hence, more randomly created complex systems (**M**) are stable given variation in γ than when $\gamma = 1$. Note that feasibility results were omitted for the table above, but are reported below.

Stability of random ecological networks

While the foundational work of May[1] applies broadly to complex networks, much attention has been given specifically to ecological networks of interacting species. In these networks, the matrix **A** is interpreted as a community matrix and each row and column is interpreted as a single species. The per capita effect that the density of any species i has on the population dynamics of species j is found in A_{ij}, meaning that **A** holds the effects of pair-wise interactions between S species[2,3]. While May's original work[1] considered only randomly assembled communities, recent work has specifically looked at more restricted ecological communities including competitive networks (all off-diagonal elements of **A** are negative), mutualist networks (all off-diagonal elements of **A** are positive), and predator-prey networks (for any pair of i and j, the effect of i on j is negative and j on i is positive, or vice versa)[2,3]. In general, competitor and mutualist networks tend to be unstable, while predator-prey networks tend to be highly stabilising[2].

I investigated competitor, mutualist, and predator-prey networks following Allesina et al.[2]. To create these networks, I first generated a random matrix **A**, then changed the elements of **A** accordingly. If **A** was a competitive network, then the sign of any positive off-diagonal elements was reversed to be negative. If **A** was a mutualist network, then the sign of any positive off-diagonal elements was reversed to be positive. And if **A** was a predator-prey network, then all i and j pairs of elements were checked; any pairs of the same sign were changed so that one was negative and the other was positive.

The number of stable $\mathbf{M} = \gamma\mathbf{A}$ systems was calculated exactly as it was for random matrices for values of S from 2 to 50 (100 in the case of the relatively more stable predator-prey interactions), except that only 100000 random **M** were generated instead of 1 million.

The following tables for restricted ecological communities can therefore be compared with the random **M** results above (but note that counts from systems with comparable probabilities of stability will be an order of magnitude lower in the tables below due to the smaller number of **M** matrices generated). As with the results above, in the tables below, `A` refers to matrices **A** when $\gamma = 1$ and `M` refers to matrices after σ^2_γ is added. The column `A_unstable` shows the number of **A** matrices that are unstable, and the column `A_stable` shows the number of **A** matrices that are stable (these two columns sum to 100000). Similarly, the column `M_unstable` shows the number of **M** matrices that are unstable and `M_stable` shows the number that are stable. The columns `A_stabilised` and `A_destabilised` show how many **A** matrices were stabilised or destabilised, respectively, by σ^2_γ.

Competition

Results for competitor interaction networks are shown below

S	A_unstable	A_stable	M_unstable	M_stable	A_stabilised	A_destabilised
2	48	99952	48	99952	0	0
3	229	99771	231	99769	0	2
4	701	99299	704	99296	0	3
5	1579	98421	1587	98413	0	8
6	3218	96782	3253	96747	6	41
7	5519	94481	5619	94381	23	123
8	9062	90938	9237	90763	77	252
9	13436	86564	13729	86271	230	523
10	18911	81089	19303	80697	505	897
11	25594	74406	25961	74039	1011	1378
12	33207	66793	33382	66618	1724	1899
13	41160	58840	41089	58911	2655	2584
14	50575	49425	49894	50106	3777	3096
15	59250	40750	57892	42108	4824	3466
16	67811	32189	65740	34260	5634	3563
17	75483	24517	73056	26944	5943	3516
18	82551	17449	79878	20122	5780	3107
19	88030	11970	85204	14796	5417	2591
20	92254	7746	89766	10234	4544	2056
21	95233	4767	93002	6998	3695	1464
22	97317	2683	95451	4549	2803	937
23	98508	1492	97122	2878	1991	605
24	99240	760	98407	1593	1216	383
25	99669	331	99082	918	739	152
26	99871	129	99490	510	452	71
27	99938	62	99732	268	240	34
28	99985	15	99888	112	108	11
29	99990	10	99951	49	46	7
30	100000	0	99981	19	19	0
31	100000	0	99993	7	7	0
32	100000	0	99996	4	4	0
33	100000	0	99998	2	2	0
34	100000	0	100000	0	0	0
...	...	...	...	...	...	...
50	100000	0	100000	0	0	0

Mutualism

Results for mutualist interaction networks are shown below

S	A_unstable	A_stable	M_unstable	M_stable	A_stabilised	A_destabilised
2	56	99944	56	99944	0	0
3	3301	96699	3301	96699	0	0
4	34446	65554	34446	65554	0	0
5	86520	13480	86520	13480	0	0
6	99683	317	99683	317	0	0
7	99998	2	99998	2	0	0
8	100000	0	100000	0	0	0
9	100000	0	100000	0	0	0
10	100000	0	100000	0	0	0
11	100000	0	100000	0	0	0
12	100000	0	100000	0	0	0

S	A_unstable	A_stable	M_unstable	M_stable	A_stabilised	A_destabilised
...	...	...	...	...	...	...
50	100000	0	100000	0	0	0

Predator-prey

Results for predator-prey interaction networks are shown below

S	A_unstable	A_stable	M_unstable	M_stable	A_stabilised	A_destabilised
2	0	100000	0	100000	0	0
3	0	100000	0	100000	0	0
4	0	100000	0	100000	0	0
5	1	99999	1	99999	0	0
6	4	99996	4	99996	0	0
7	2	99998	2	99998	0	0
8	5	99995	5	99995	0	0
9	20	99980	21	99979	0	1
10	20	99980	22	99978	0	2
11	38	99962	39	99961	0	1
12	64	99936	66	99934	0	2
13	87	99913	91	99909	0	4
14	157	99843	159	99841	0	2
15	215	99785	227	99773	0	12
16	293	99707	310	99690	0	17
17	383	99617	408	99592	0	25
18	443	99557	473	99527	3	33
19	642	99358	675	99325	4	37
20	836	99164	887	99113	7	58
21	1006	98994	1058	98942	10	62
22	1153	98847	1228	98772	20	95
23	1501	98499	1593	98407	30	122
24	1841	98159	1996	98004	40	195
25	2146	97854	2316	97684	58	228
26	2643	97357	2809	97191	119	285
27	3034	96966	3258	96742	158	382
28	3690	96310	3928	96072	201	439
29	4257	95743	4532	95468	290	565
30	4964	95036	5221	94779	424	681
31	5627	94373	5978	94022	452	803
32	6543	93457	6891	93109	666	1014
33	7425	92575	7777	92223	818	1170
34	8540	91460	8841	91159	1071	1372
35	9526	90474	9842	90158	1337	1653
36	10617	89383	10891	89109	1624	1898
37	12344	87656	12508	87492	2021	2185
38	13675	86325	13877	86123	2442	2644
39	15264	84736	15349	84651	2870	2955
40	17026	82974	17053	82947	3363	3390
41	18768	81232	18614	81386	3905	3751
42	20791	79209	20470	79530	4579	4258
43	23150	76850	22754	77246	5217	4821
44	25449	74551	24184	75816	6285	5020
45	27702	72298	26464	73536	6754	5516

S	A_unstable	A_stable	M_unstable	M_stable	A_stabilised	A_destabilised
46	30525	69475	28966	71034	7646	6087
47	32832	67168	31125	68875	8487	6780
48	36152	63848	33865	66135	9479	7192
49	38714	61286	36242	63758	10125	7653
50	41628	58372	38508	61492	11036	7916
51	44483	55517	41023	58977	11704	8244
52	48134	51866	44287	55713	12573	8726
53	51138	48862	46721	53279	13223	8806
54	54261	45739	49559	50441	13757	9055
55	57647	42353	52403	47597	14324	9080
56	60630	39370	55293	44707	14669	9332
57	63647	36353	57787	42213	15103	9243
58	66961	33039	60439	39561	15450	8928
59	69968	30032	63708	36292	15246	8986
60	72838	27162	66270	33730	15177	8609
61	75609	24391	68873	31127	15006	8270
62	77999	22001	71318	28682	14538	7857
63	80616	19384	73517	26483	14510	7411
64	83089	16911	76209	23791	13784	6904
65	85150	14850	78086	21914	13412	6348
66	86908	13092	80437	19563	12477	6006
67	88671	11329	82379	17621	11718	5426
68	90537	9463	84483	15517	10878	4824
69	91969	8031	86233	13767	10033	4297
70	93181	6819	87914	12086	9070	3803
71	94330	5670	89200	10800	8401	3271
72	95324	4676	90833	9167	7359	2868
73	96143	3857	91805	8195	6726	2388
74	96959	3041	93065	6935	5900	2006
75	97543	2457	93987	6013	5222	1666
76	97969	2031	94900	5100	4481	1412
77	98497	1503	95756	4244	3809	1068
78	98744	1256	96442	3558	3269	967
79	99045	955	96942	3058	2837	734
80	99276	724	97528	2472	2329	581
81	99481	519	97996	2004	1894	409
82	99556	444	98321	1679	1597	362
83	99691	309	98722	1278	1227	258
84	99752	248	98943	1057	1015	206
85	99833	167	99144	856	837	148
86	99895	105	99346	654	642	93
87	99925	75	99461	539	530	66
88	99945	55	99566	434	428	49
89	99976	24	99675	325	324	23
90	99977	23	99756	244	243	22
91	99982	18	99839	161	155	12
92	99988	12	99865	135	135	12
93	99994	6	99885	115	115	6
94	99993	7	99911	89	88	6
95	99998	2	99953	47	47	2
96	99999	1	99965	35	35	1
97	99999	1	99979	21	21	1

S	A_unstable	A_stable	M_unstable	M_stable	A_stabilised	A_destabilised
98	100000	0	99973	27	27	0
99	100000	0	99984	16	16	0
100	100000	0	99989	11	11	0

Overall, as expected[2], predator-prey communities are relatively stable while mutualist communties are highly unstable. But interestingly, while σ^2_γ stabilises predator-prey and competitor communities, it does not stabilise mutualist communities. This is unsurprising because purely mutualist communities are characterised by a very positive[2] leading $\Re(\lambda)$, and it is highly unlikely that σ^2_γ alone will shift all real parts of eigenvalues to negative values.

Sensitivity of connectance (C) values

In the main text, for simplicity, I assumed connectance values of $C = 1$, meaning that all off-diagonal elements of a matrix **M** were potentially nonzero and sampled from a normal distribution $\mathcal{N}(0, \sigma^2_A)$ where $\sigma_A = 0.4$. Here I present four tables showing the number of stable communities given $C = \{0.3, 0.5, 0.7, 0.9\}$. In all cases, uniform variation in component response rate ($\gamma \sim \mathcal{U}(0, 2)$) led to a higher number of stable communities than when γ did not vary ($\gamma = 1$). In contrast to the main text, 100000 rather than 1 million **M** were simulated. As with the results on stability with increasing S shown above, in the tables below `A` refers to **A** matrices when $\gamma = 1$, and `M` refers to **M** matrices after σ^2_γ is added. The column `A_unstable` shows the number of **A** matrices that are unstable, and the column `A_stable` shows the number of **A** matrices that are stable (these two columns sum to 100000). Similarly, the column `M_unstable` shows the number of **M** matrices that are unstable and `M_stable` shows the number that are stable. The columns `A_stabilised` and `A_destabilised` show how many **A** matrices were stabilised or destabilised, respectively, by σ^2_γ.

Connectance C = 0.3

S	A_unstable	A_stable	M_unstable	M_stable	A_stabilised	A_destabilised
2	5	99995	5	99995	0	0
3	6	99994	6	99994	0	0
4	24	99976	24	99976	0	0
5	59	99941	59	99941	0	0
6	98	99902	98	99902	0	0
7	160	99840	161	99839	0	1
8	290	99710	293	99707	0	3
9	430	99570	434	99566	0	4
10	648	99352	653	99347	1	6
11	946	99054	957	99043	0	11
12	1392	98608	1415	98585	4	27
13	2032	97968	2065	97935	5	38
14	2627	97373	2688	97312	10	71
15	3588	96412	3647	96353	35	94
16	5019	94981	5124	94876	51	156
17	6512	93488	6673	93327	79	240
18	8444	91556	8600	91400	165	321
19	10416	89584	10667	89333	244	495
20	13254	86746	13477	86523	425	648
21	16248	83752	16481	83519	642	875
22	19497	80503	19719	80281	929	1151
23	23654	76346	23776	76224	1368	1490
24	28485	71515	28389	71611	1914	1818

S	A_unstable	A_stable	M_unstable	M_stable	A_stabilised	A_destabilised
25	32774	67226	32483	67517	2428	2137
26	38126	61874	37411	62589	3221	2506
27	43435	56565	42418	57582	3828	2811
28	49333	50667	47840	52160	4565	3072
29	55389	44611	53381	46619	5329	3321
30	60826	39174	58388	41612	5918	3480
31	66820	33180	64043	35957	6345	3568
32	72190	27810	69036	30964	6685	3531
33	77053	22947	73587	26413	6826	3360
34	81816	18184	78157	21843	6673	3014
35	85651	14349	82041	17959	6383	2773
36	88985	11015	85657	14343	5721	2393
37	92072	7928	88805	11195	5180	1913
38	94329	5671	91444	8556	4451	1566
39	95912	4088	93295	6705	3804	1187
40	97232	2768	95201	4799	2967	936
41	98179	1821	96506	3494	2356	683
42	98826	1174	97489	2511	1786	449
43	99275	725	98312	1688	1251	288
44	99583	417	98872	1128	903	192
45	99776	224	99339	661	576	139
46	99865	135	99518	482	413	66
47	99938	62	99744	256	226	32
48	99956	44	99824	176	151	19
49	99980	20	99914	86	85	19
50	99993	7	99950	50	46	3
51	99998	2	99971	29	28	1
52	99998	2	99986	14	14	2
53	99999	1	99992	8	7	0
54	100000	0	99997	3	3	0
55	100000	0	99999	1	1	0
56	100000	0	99998	2	2	0
57	100000	0	99999	1	1	0
58	100000	0	100000	0	0	0
...	...	...	...	...	...	...
100	100000	0	100000	0	0	0

Connectance C = 0.5

S	A_unstable	A_stable	M_unstable	M_stable	A_stabilised	A_destabilised
2	7	99993	7	99993	0	0
3	32	99968	32	99968	0	0
4	122	99878	122	99878	0	0
5	320	99680	321	99679	0	1
6	667	99333	673	99327	0	6
7	1233	98767	1252	98748	0	19
8	2123	97877	2156	97844	3	36
9	3415	96585	3471	96529	16	72
10	5349	94651	5450	94550	30	131
11	7990	92010	8185	91815	81	276
12	11073	88927	11301	88699	219	447

S	A_unstable	A_stable	M_unstable	M_stable	A_stabilised	A_destabilised
13	14971	85029	15204	84796	445	678
14	19754	80246	19992	80008	764	1002
15	25020	74980	25239	74761	1185	1404
16	30860	69140	30938	69062	1902	1980
17	37844	62156	37562	62438	2758	2476
18	44909	55091	44251	55749	3595	2937
19	52322	47678	51011	48989	4573	3262
20	60150	39850	58295	41705	5382	3527
21	67147	32853	64895	35105	5925	3673
22	74177	25823	71358	28642	6310	3491
23	80297	19703	77034	22966	6507	3244
24	85372	14628	82039	17961	6209	2876
25	89719	10281	86539	13461	5562	2382
26	92947	7053	90141	9859	4707	1901
27	95436	4564	92950	7050	3844	1358
28	97196	2804	95171	4829	2999	974
29	98300	1700	96842	3158	2115	657
30	99103	897	98033	1967	1466	396
31	99502	498	98665	1335	1068	231
32	99745	255	99185	815	696	136
33	99881	119	99572	428	375	66
34	99955	45	99788	212	191	24
35	99979	21	99900	100	95	16
36	99995	5	99950	50	50	5
37	99997	3	99970	30	28	1
38	99998	2	99986	14	13	1
39	99999	1	99991	9	9	1
40	100000	0	100000	0	0	0
41	100000	0	99999	1	1	0
42	100000	0	99999	1	1	0
43	100000	0	100000	0	0	0
...	...	...	...	...	...	...
50	100000	0	100000	0	0	0

Connectance C = 0.7

S	A_unstable	A_stable	M_unstable	M_stable	A_stabilised	A_destabilised
2	7	99993	7	99993	0	0
3	106	99894	106	99894	0	0
4	395	99605	397	99603	0	2
5	1117	98883	1123	98877	0	6
6	2346	97654	2367	97633	6	27
7	4314	95686	4388	95612	16	90
8	7327	92673	7456	92544	61	190
9	11514	88486	11792	88208	150	428
10	16247	83753	16584	83416	415	752
11	22481	77519	22759	77241	884	1162
12	29459	70541	29729	70271	1548	1818
13	37631	62369	37567	62433	2419	2355
14	46317	53683	45696	54304	3548	2927
15	54945	45055	53695	46305	4671	3421

S	A_unstable	A_stable	M_unstable	M_stable	A_stabilised	A_destabilised
16	63683	36317	61643	38357	5567	3527
17	72004	27996	69375	30625	6124	3495
18	79220	20780	76158	23842	6413	3351
19	85286	14714	82283	17717	5982	2979
20	90240	9760	87181	12819	5398	2339
21	93676	6324	91077	8923	4468	1869
22	96203	3797	94045	5955	3425	1267
23	97866	2134	96161	3839	2496	791
24	98842	1158	97633	2367	1713	504
25	99433	567	98630	1370	1079	276
26	99760	240	99259	741	655	154
27	99895	105	99576	424	377	58
28	99950	50	99790	210	194	34
29	99981	19	99915	85	80	14
30	99994	6	99952	48	47	5
31	99998	2	99972	28	28	2
32	99999	1	99992	8	8	1
33	100000	0	99997	3	3	0
34	100000	0	99999	1	1	0
35	100000	0	100000	0	0	0
...	...	...	...	...	...	...
50	100000	0	100000	0	0	0

Connectance C = 0.9

S	A_unstable	A_stable	M_unstable	M_stable	A_stabilised	A_destabilised
2	14	99986	14	99986	0	0
3	240	99760	240	99760	0	0
4	1008	98992	1016	98984	0	8
5	2708	97292	2729	97271	2	23
6	5669	94331	5755	94245	13	99
7	9848	90152	10057	89943	91	300
8	15903	84097	16201	83799	336	634
9	22707	77293	23110	76890	765	1168
10	30796	69204	31122	68878	1526	1852
11	40224	59776	40082	59918	2649	2507
12	49934	50066	49288	50712	3773	3127
13	60138	39862	58803	41197	4984	3649
14	69100	30900	67110	32890	5755	3765
15	77607	22393	74884	25116	6273	3550
16	84663	15337	81780	18220	5975	3092
17	90075	9925	87290	12710	5209	2424
18	93944	6056	91419	8581	4271	1746
19	96650	3350	94530	5470	3287	1167
20	98160	1840	96698	3302	2191	729
21	99111	889	98133	1867	1389	411
22	99588	412	98905	1095	903	220
23	99837	163	99480	520	452	95
24	99932	68	99744	256	228	40
25	99976	24	99863	137	133	20
26	99995	5	99950	50	49	4

S	A_unstable	A_stable	M_unstable	M_stable	A_stabilised	A_destabilised
27	99996	4	99986	14	13	3
28	100000	0	99993	7	7	0
29	100000	0	99996	4	4	0
30	100000	0	99998	2	2	0
31	100000	0	100000	0	0	0
...	...	...	...	...	...	...
50	100000	0	100000	0	0	0

Sensitivity of interaction strength (σ_A) values

Results below show stability results given varying interaction strengths (σ_A) for $C = 0.05$ (note that system size S values are larger and increase by 10 with increasing rows). In the tables below (as above), `A` and `M` refers to matrices for $\gamma = 1$ and σ_γ^2, respectively.

Interaction strength $\sigma_{\mathbf{A}} = 0.3$

S	A_unstable	A_stable	M_unstable	M_stable	A_stabilised	A_destabilised
10	0	100000	0	100000	0	0
20	0	100000	0	100000	0	0
30	0	100000	0	100000	0	0
40	0	100000	0	100000	0	0
50	0	100000	0	100000	0	0
60	2	99998	2	99998	0	0
70	4	99996	4	99996	0	0
80	6	99994	6	99994	0	0
90	5	99995	5	99995	0	0
100	11	99989	11	99989	0	0
110	12	99988	13	99987	0	1
120	23	99977	23	99977	0	0
130	40	99960	40	99960	0	0
140	62	99938	65	99935	0	3
150	162	99838	165	99835	0	3
160	325	99675	329	99671	2	6
170	829	99171	851	99149	6	28
180	1817	98183	1860	98140	31	74
190	3927	96073	3989	96011	143	205
200	8084	91916	8048	91952	557	521
210	15558	84442	15147	84853	1534	1123
220	26848	73152	25342	74658	3625	2119
230	43386	56614	39535	60465	6992	3141
240	62734	37266	56684	43316	9815	3765
250	80128	19872	73080	26920	10128	3080
260	92206	7794	86619	13381	7490	1903
270	97946	2054	94824	5176	3797	675
280	99659	341	98534	1466	1265	140
290	99962	38	99696	304	281	15
300	99994	6	99964	36	34	4

Interaction strength $\sigma_{\mathbf{A}} = 0.4$

S	A_unstable	A_stable	M_unstable	M_stable	A_stabilised	A_destabilised
10	3	99997	3	99997	0	0
20	15	99985	15	99985	0	0
30	48	99952	48	99952	0	0
40	85	99915	85	99915	0	0
50	163	99837	163	99837	0	0
60	280	99720	282	99718	0	2
70	561	99439	566	99434	3	8
80	1009	98991	1029	98971	6	26
90	2126	97874	2175	97825	31	80
100	4580	95420	4653	95347	142	215
110	9540	90460	9632	90368	465	557
120	19090	80910	18668	81332	1676	1254
130	35047	64953	33220	66780	4172	2345
140	56411	43589	52439	47561	7297	3325
150	78003	21997	72574	27426	8477	3048
160	92678	7322	88438	11562	5901	1661
170	98614	1386	96670	3330	2397	453
180	99839	161	99418	582	499	78
190	99990	10	99945	55	52	7
200	100000	0	99995	5	5	0
210	100000	0	100000	0	0	0
...	...	...	...	...	...	...
300	100000	0	100000	0	0	0

Interaction strength $\sigma_A = 0.5$

S	A_unstable	A_stable	M_unstable	M_stable	A_stabilised	A_destabilised
10	36	99964	36	99964	0	0
20	195	99805	195	99805	0	0
30	519	99481	523	99477	0	4
40	1096	98904	1101	98899	2	7
50	2375	97625	2397	97603	9	31
60	4898	95102	4968	95032	83	153
70	10841	89159	10916	89084	432	507
80	22281	77719	21988	78012	1622	1329
90	42010	57990	39998	60002	4458	2446
100	67289	32711	63098	36902	7153	2962
110	88137	11863	84023	15977	6108	1994
120	97678	2322	95557	4443	2740	619
130	99795	205	99304	696	578	87
140	99989	11	99948	52	49	8
150	100000	0	100000	0	0	0
...	...	...	...	...	...	...
300	100000	0	100000	0	0	0

Interaction strength $\sigma_A = 0.6$

S	A_unstable	A_stable	M_unstable	M_stable	A_stabilised	A_destabilised
10	162	99838	162	99838	0	0
20	798	99202	799	99201	0	1

S	A_unstable	A_stable	M_unstable	M_stable	A_stabilised	A_destabilised
30	2273	97727	2289	97711	6	22
40	5259	94741	5298	94702	70	109
50	12084	87916	12054	87946	446	416
60	26072	73928	25511	74489	1810	1249
70	50121	49879	47747	52253	4748	2374
80	77806	22194	73810	26190	6421	2425
90	94862	5138	92069	7931	3842	1049
100	99527	473	98822	1178	870	165
110	99984	16	99912	88	80	8
120	100000	0	99998	2	2	0
130	100000	0	100000	0	0	0
...	...	...	...	...	...	...
300	100000	0	100000	0	0	0

Sensitivity of distribution of γ

In the main text, I considered a uniform distribution of component response rates $\gamma \sim \mathcal{U}(0, 2)$. The number of unstable and stable **M** matrices are reported in a table above across different values of S. Here I show complementary results for three different distributions including an exponential, beta, and gamma distribution of γ values. The shape of these distributions is shown in the figure below.

Distributions of component response rate (γ) values in complex systems. The stabilities of simulated complex systems with these γ distributions are compared to identical systems in which $\gamma = 1$ across different system sizes (S; i.e., component numbers) given a unit γ standard deviation ($\sigma_\gamma = 1$) for b-d. Distributions are as follows: (a) uniform, (b) exponential, (c) beta ($\alpha = 0.5$ and $\beta = 0.5$), and (d) gamma ($k = 2$ and $\theta = 2$). Each panel shows 1 million randomly generated γ values.

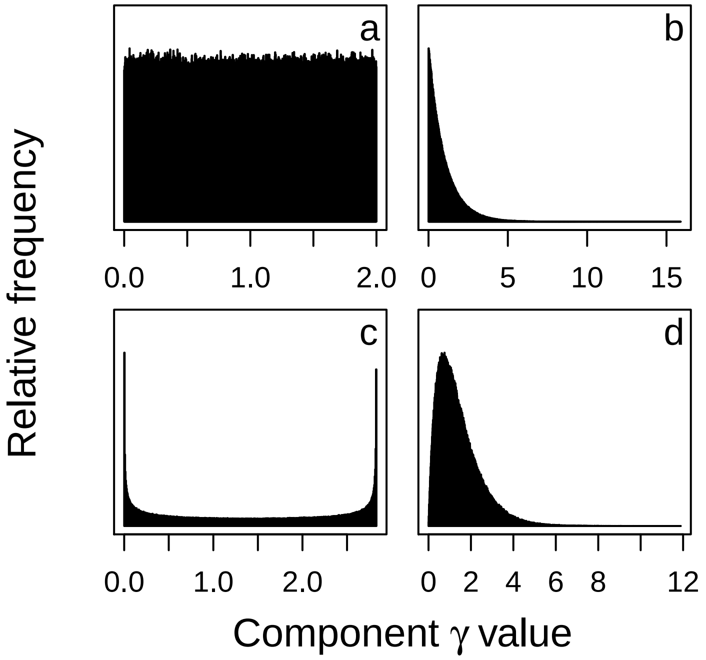

The stability of **A** versus **M** was investigated for each of the distributions of γ shown in panels b-d above. The table below shows the number of **A** versus **M** that were stable for the exponential (exp), beta, and gamma distributions.

S	exp_A	exp_M	beta_A	beta_M	gamma_A	gamma_M
2	99965	99965	99974	99974	99977	99977
3	99636	99635	99650	99648	99628	99628
4	98576	98564	98482	98470	98508	98492
5	96053	95971	96156	96096	96068	96004
6	92036	91867	92104	91927	92233	92029
7	86667	86333	86456	86070	86604	86161
8	79670	79153	79392	78822	79393	78771
9	71389	70911	70998	70529	71070	70548
10	61674	61609	61794	61586	61265	61093
11	51150	51935	51352	51924	51313	51951
12	41209	42925	40954	42670	40708	42183
13	30827	33462	30969	33770	31046	33522
14	22203	25767	22208	25629	22342	25435
15	15003	18877	15206	18913	15025	18464
16	9613	13372	9504	13357	9418	12737
17	5579	8967	5570	8976	5719	8487
18	3104	5833	3048	5853	3060	5447
19	1516	3578	1553	3633	1600	3185
20	717	2067	799	2179	769	1862
21	312	1196	310	1200	331	1039
22	129	643	128	654	135	510
23	48	321	48	359	57	242
24	11	161	19	159	20	120
25	1	59	5	81	7	45
26	0	30	0	48	0	22
27	0	10	0	16	0	6
28	1	3	2	2	0	3
29	0	2	0	0	0	0
30	0	0	0	1	0	0
31	0	0	0	1	0	0
32	0	0	0	0	0	0
...	...	...	...	...	...	...
50	0	0	0	0	0	0

In comparison to the uniform distribution (a), proportionally fewer random systems are found with the exponential distribution (b), while more are found with the beta (c) and gamma (d) distributions.

Stability of structured networks

I tested the stability of one million random, small-world, scale-free, and cascade food web networks for different network parameters. Each of these networks is structured differently. In the main text, the random networks and cascade food webs that I built were saturated ($C = 1$), meaning that every component was connected to, and interacted with, every other component (see immediately below).

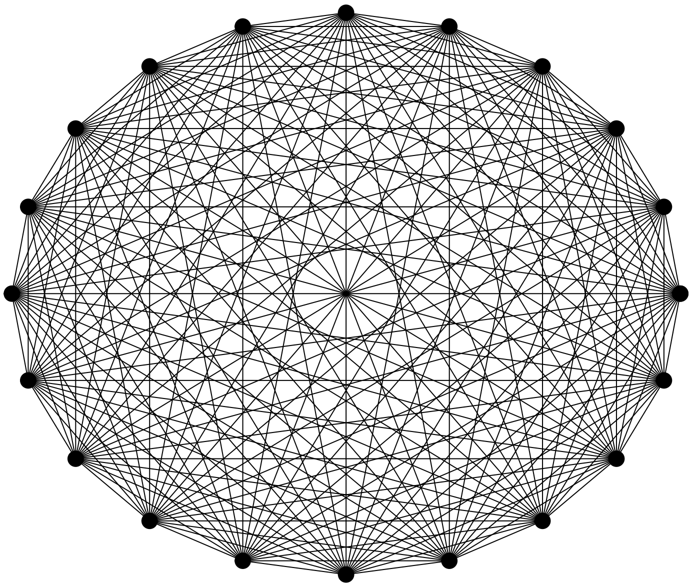

Small-world networks, in contrast, are not saturated. They are instead defined by components that interact mostly with other closely neighbouring components, but have a proportion of interactions (β) that are instead between non-neighbours[4]. Two small-world networks are shown below.

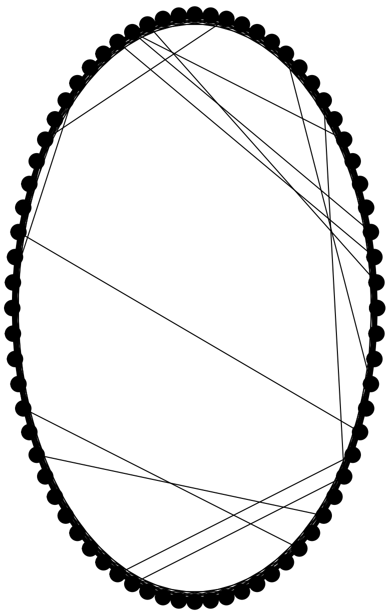

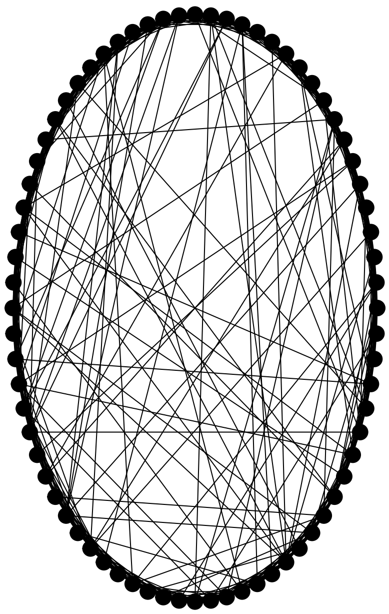

The small-world network on the left shows a system in which $\beta = 0.01$, while the small-world network on the right shows one in which $\beta = 0.1$. At the extremes of $\beta = 0$ and $\beta = 1$, networks are regular and random, respectively. The table below shows how σ^2_γ affects stability in small world networks across different values of S and β.

beta	S	A_unstable	A_stable	M_unstable	M_stable	complex_A	complex_M	C
0.00	24	17388	982612	17446	982554	0.5748066	0.6582632	0.1304348
0.00	48	258024	741976	260579	739421	0.8073918	0.9294192	0.1063830
0.00	72	715036	284964	722639	277361	0.9860840	1.1364805	0.0985915
0.00	96	961434	38566	962788	37212	1.1369395	1.3110263	0.0947368

beta	S	A_unstable	A_stable	M_unstable	M_stable	complex_A	complex_M	C
0.00	120	999008	992	998857	1143	1.2700387	1.4649832	0.0924370
0.00	144	999997	3	999994	6	1.3903192	1.6041216	0.0909091
0.00	168	1000000	0	1000000	0	1.5010334	1.7320676	0.0898204
0.01	24	17673	982327	17720	982280	0.5747156	0.6581503	0.1304319
0.01	48	255038	744962	257647	742353	0.8073388	0.9292952	0.1063800
0.01	72	708892	291108	716829	283171	0.9859457	1.1363940	0.0985884
0.01	96	960635	39365	961876	38124	1.1370640	1.3112193	0.0947337
0.01	120	999040	960	998794	1206	1.2698715	1.4648280	0.0924338
0.01	144	999997	3	999994	6	1.3901601	1.6039285	0.0909060
0.01	168	1000000	0	1000000	0	1.5009490	1.7319739	0.0898173
0.10	24	20382	979618	20455	979545	0.5742520	0.6573563	0.1302974
0.10	48	237747	762253	240370	759630	0.8066604	0.9284434	0.1062311
0.10	72	679874	320126	685575	314425	0.9849695	1.1352553	0.0984349
0.10	96	961984	38016	960128	39872	1.1358912	1.3097957	0.0945788
0.10	120	999546	454	999275	725	1.2687142	1.4634587	0.0922779
0.10	144	1000000	0	1000000	0	1.3890356	1.6025900	0.0907489
0.10	168	1000000	0	1000000	0	1.4994818	1.7302649	0.0896598
0.25	24	23654	976346	23775	976225	0.5722185	0.6546853	0.1296712
0.25	48	228318	771682	231208	768792	0.8033257	0.9244966	0.1055259
0.25	72	666982	333018	669104	330896	0.9808676	1.1304109	0.0977066
0.25	96	966456	33544	961545	38455	1.1307841	1.3039452	0.0938392
0.25	120	999749	251	999507	493	1.2632327	1.4571506	0.0915316
0.25	144	1000000	0	1000000	0	1.3827642	1.5953248	0.0899987
0.25	168	1000000	0	1000000	0	1.4926700	1.7224506	0.0889064
1.00	24	26331	973669	26478	973522	0.5561013	0.6356655	0.1249651
1.00	48	211199	788801	214154	785846	0.7720342	0.8881302	0.0991370
1.00	72	613621	386379	615771	384229	0.9394912	1.0825566	0.0908153
1.00	96	943191	56809	936396	63604	1.0812364	1.2466510	0.0867047
1.00	120	999157	843	998396	1604	1.2065026	1.3916458	0.0842561
1.00	144	1000000	0	999997	3	1.3199179	1.5227509	0.0826325
1.00	168	1000000	0	1000000	0	1.4243560	1.6434386	0.0814738

In the above, the complexity of **A** and **M**, and the mean C, are also shown. For similar magnitudes of complexity as in random networks of $\sigma\sqrt{SC} \gtrapprox 1.26$, variation in γ typically results in more stable than unstable systems.

Scale-free networks are also not saturated, but are defined by an interaction frequency distribution that follows a power law. In other words, a small number of components interact with many other components, while most components interact with only a small number of other components. Scale-free networks can be built by adding new components, one by one, to an existing system, with each newly added component interacting with a randomly selected subset of m existing components[5]. The network on the left below shows an example of a scale-free network in which $m = 3$. The histogram on the right shows the number of other components with which each component interacts.

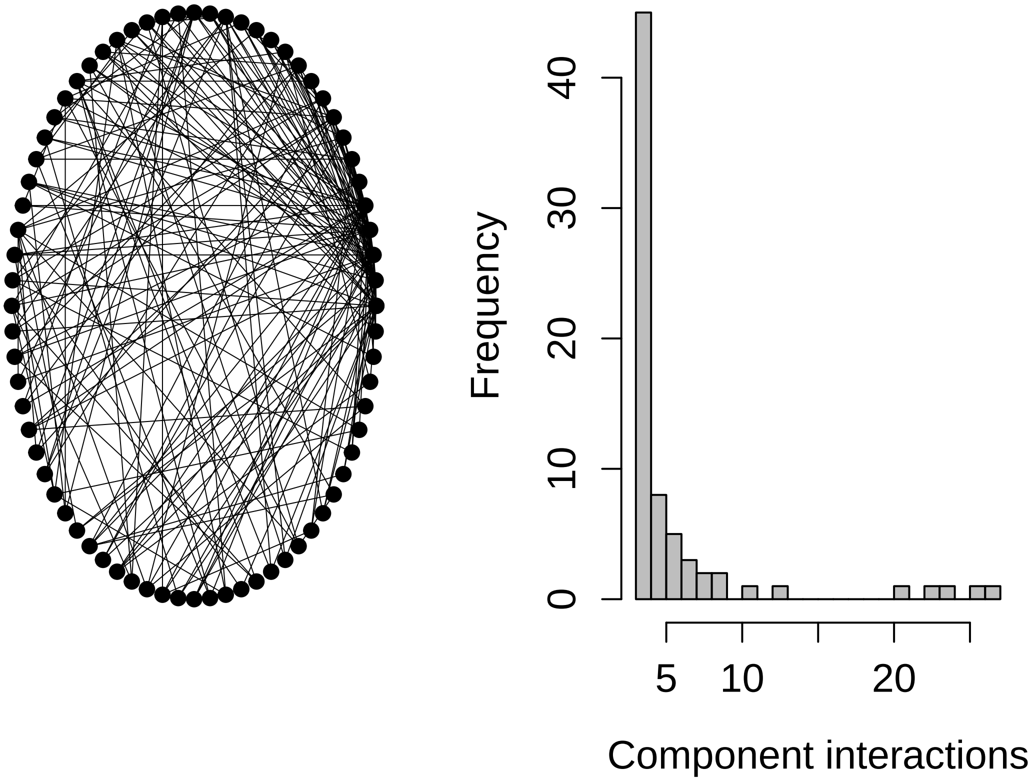

The table below shows how σ_γ^2 affects stability across different scale-free networks with different S and m values.

m	S	A_unstable	A_stable	M_unstable	M_stable	complex_A	complex_M	C
2	24	152791	847209	156034	843966	0.7891257	0.9034663	0.1648551
3	24	320481	679519	326351	673649	0.9566487	1.0967499	0.2409420
4	24	504433	495567	504826	495174	1.0922870	1.2532761	0.3134058
5	24	670676	329324	660426	339574	1.2073054	1.3857169	0.3822464
6	24	798637	201363	779345	220655	1.3067095	1.5004508	0.4474638
7	24	884082	115918	862215	137785	1.3942577	1.6013368	0.5090580
8	24	936190	63810	915630	84370	1.4722315	1.6908563	0.5670290
9	24	964868	35132	948297	51703	1.5414455	1.7707292	0.6213768
10	24	981460	18540	967911	32089	1.6030044	1.8417459	0.6721014
11	24	989838	10162	980232	19768	1.6586511	1.9059313	0.7192029
12	24	994393	5607	987436	12564	1.7081503	1.9628898	0.7626812
2	48	303963	696037	310053	689947	0.7946875	0.9132519	0.0828901
3	48	577855	422145	579996	420004	0.9685494	1.1141445	0.1227837
4	48	810001	189999	799132	200868	1.1122992	1.2799335	0.1617908
5	48	938004	61996	924613	75387	1.2369960	1.4236817	0.1999113
6	48	984975	15025	976433	23567	1.3478291	1.5514420	0.2371454
7	48	997160	2840	994005	5995	1.4473792	1.6663763	0.2734929
8	48	999584	416	998590	1410	1.5385445	1.7716359	0.3089539
9	48	999955	45	999707	293	1.6227742	1.8687074	0.3435284
10	48	999992	8	999939	61	1.7006157	1.9583879	0.3772163
11	48	999999	1	999990	10	1.7731759	2.0420990	0.4100177
12	48	1000000	0	999999	1	1.8410402	2.1203112	0.4419326
2	72	427243	572757	434600	565400	0.7964226	0.9166566	0.0553599
3	72	741345	258655	739020	260980	0.9723446	1.1195788	0.0823552
4	72	931043	68957	921145	78855	1.1188220	1.2888100	0.1089593

m	S	A_unstable	A_stable	M_unstable	M_stable	complex_A	complex_M	C
5	72	989644	10356	984372	15628	1.2466268	1.4361875	0.1351721
6	72	999131	869	997914	2086	1.3604666	1.5674966	0.1609937
7	72	999946	54	999804	196	1.4642496	1.6872501	0.1864241
8	72	999999	1	999988	12	1.5596340	1.7974044	0.2114632
9	72	1000000	0	999999	1	1.6482181	1.8994441	0.2361111
10	72	1000000	0	1000000	0	1.7307859	1.9947150	0.2603678
11	72	1000000	0	1000000	0	1.8086766	2.0847262	0.2842332
12	72	1000000	0	1000000	0	1.8817533	2.1689764	0.3077074
2	96	527633	472367	535188	464812	0.7974024	0.9183557	0.0415570
3	96	842274	157726	837756	162244	0.9741293	1.1224709	0.0619518
4	96	975834	24166	969478	30522	1.1220115	1.2931371	0.0821272
5	96	998391	1609	996991	3009	1.2511287	1.4422331	0.1020833
6	96	999955	45	999838	162	1.3669903	1.5757699	0.1218202
7	96	999999	1	999996	4	1.4725862	1.6977057	0.1413377
8	96	1000000	0	1000000	0	1.5699145	1.8099762	0.1606360
9	96	1000000	0	1000000	0	1.6606162	1.9146804	0.1797149
10	96	1000000	0	1000000	0	1.7457971	2.0129344	0.1985746
11	96	1000000	0	1000000	0	1.8260368	2.1055559	0.2172149
12	96	1000000	0	1000000	0	1.9018608	2.1929362	0.2356360
2	120	609563	390437	616036	383964	0.7979355	0.9194404	0.0332633
3	120	904064	95936	899040	100960	0.9753815	1.1243251	0.0496499
4	120	991710	8290	988410	11590	1.1239922	1.2957520	0.0658964
5	120	999781	219	999477	523	1.2539362	1.4458518	0.0820028
6	120	999999	1	999981	19	1.3707937	1.5806987	0.0979692
7	120	1000000	0	999999	1	1.4775366	1.7038860	0.1137955
8	120	1000000	0	1000000	0	1.5762636	1.8177236	0.1294818
9	120	1000000	0	1000000	0	1.6680647	1.9238257	0.1450280
10	120	1000000	0	1000000	0	1.7545110	2.0233838	0.1604342
11	120	1000000	0	1000000	0	1.8363882	2.1178385	0.1757003
12	120	1000000	0	1000000	0	1.9135798	2.2069806	0.1908263

As in small-world networks, the mean C is shown, along with the mean complexities of $\mathbf{A}$ and $\mathbf{M}$. Like all other networks, σ_γ^2 increases the stability of scale-free networks given sufficiently high complexity.

Cascade food webs are saturated, and similar to predator-prey random networks. What distinguishes them from predator-prey networks is that cascade food webs are also defined by intactions in which components are ranked such that if the rank of $i > j$, then $A_{ij} < 0$ and $A_{ji} > 0$[6,7]. In other words, if interpreting components as ecological species, species can only feed off of a species of lower rank. The table below shows how σ_γ^2 affects stability across system sizes in cascade food webs.

S	A_unstable	A_stable	M_unstable	M_stable	complex_A	complex_M
2	0	1000000	0	1000000	0.6378839	0.6381485
3	1	999999	1	999999	0.7055449	0.7525143
4	2	999998	2	999998	0.8060500	0.8826100
5	17	999983	17	999983	0.8974749	0.9967594
6	42	999958	43	999957	0.9821323	1.0999762
7	124	999876	124	999876	1.0600906	1.1938910
8	303	999697	309	999691	1.1329713	1.2807302
9	653	999347	661	999339	1.2009135	1.3616372
10	1401	998599	1413	998587	1.2661142	1.4387567
11	2534	997466	2566	997434	1.3276636	1.5113096
12	4514	995486	4597	995403	1.3865754	1.5804005

S	A_unstable	A_stable	M_unstable	M_stable	complex_A	complex_M
13	7570	992430	7722	992278	1.4424479	1.6462780
14	12223	987777	12502	987498	1.4970134	1.7102322
15	18433	981567	18879	981121	1.5498812	1.7719564
16	26973	973027	27712	972288	1.6002970	1.8310447
17	38272	961728	39499	960501	1.6494195	1.8884211
18	52397	947603	54099	945901	1.6975099	1.9443860
19	69986	930014	72342	927658	1.7439233	1.9987398
20	92851	907149	95776	904224	1.7893524	2.0514394
21	117487	882513	121095	878905	1.8335974	2.1030121
22	147852	852148	151989	848011	1.8761874	2.1527108
23	183501	816499	187888	812112	1.9186092	2.2019827
24	222592	777408	226021	773979	1.9591518	2.2491948
25	267691	732309	269822	730178	1.9999089	2.2963949
26	316090	683910	316371	683629	2.0396325	2.3427211
27	369830	630170	366550	633450	2.0785319	2.3879356
28	426407	573593	419136	580864	2.1169703	2.4324407
29	485068	514932	473666	526334	2.1545265	2.4759539
30	544300	455700	527568	472432	2.1912376	2.5187795
31	605803	394197	584385	415615	2.2271037	2.5603818
32	664689	335311	638047	361953	2.2626270	2.6016360
33	718848	281152	689172	310828	2.2979241	2.6424881
34	770790	229210	737639	262361	2.3327303	2.6828460
35	817531	182469	783112	216888	2.3666720	2.7221952
36	858750	141250	823548	176452	2.3998286	2.7608037
37	893017	106983	859194	140806	2.4332806	2.7994470
38	921268	78732	890177	109823	2.4658414	2.8372307
39	943551	56449	915655	84345	2.4974678	2.8741350
40	961088	38912	936883	63117	2.5301278	2.9116114
41	973664	26336	953645	46355	2.5616210	2.9481298
42	982829	17171	967044	32956	2.5925309	2.9841081
43	989464	10536	977033	22967	2.6228949	3.0191690
44	993622	6378	984470	15530	2.6534626	3.0548439
45	996221	3779	989678	10322	2.6832092	3.0890543
46	997963	2037	993318	6682	2.7130588	3.1236201
47	998818	1182	995957	4043	2.7423480	3.1575904
48	999422	578	997446	2554	2.7714223	3.1912463
49	999746	254	998532	1468	2.7999596	3.2244020
50	999864	136	999132	868	2.8285547	3.2574510
51	999934	66	999561	439	2.8566907	3.2900943
52	999970	30	999761	239	2.8844703	3.3222721
53	999985	15	999873	127	2.9122645	3.3544290
54	999999	1	999935	65	2.9395400	3.3859103
55	1000000	0	999971	29	2.9665996	3.4173273
56	999999	1	999988	12	2.9936263	3.4486027
57	1000000	0	999989	11	3.0199283	3.4789408
58	1000000	0	999998	2	3.0460952	3.5094530
59	1000000	0	999999	1	3.0728115	3.5401634
60	1000000	0	1000000	0	3.0983367	3.5698067

Cascade food webs are more likely to be stable than small-world or scale-free networks at equivalent magnitudes of complexity (note $C = 1$ for all above rows). A higher number of stable $\mathbf{M}$ than $\mathbf{A}$ was found given $S \geq 27$.

Feasibility of complex systems

When feasibility was evaluated with and without variation in γ, there was no increase in stability for **M** where γ varied as compared to where $\gamma = 1$. Results below illustrate this result, which was general to all other simulations performed.

S	A_infeasible	A_feasible	M_infeasible	M_feasible	A_made_feasible	A_made_infeasible
2	749978	250022	749942	250058	35552	35516
3	874519	125481	874296	125704	36803	36580
4	937192	62808	937215	62785	26440	26463
5	968776	31224	968639	31361	16319	16182
6	984313	15687	984463	15537	9006	9156
7	992149	7851	992161	7839	4991	5003
8	996124	3876	996103	3897	2644	2623
9	998014	1986	998027	1973	1361	1374
10	999031	969	999040	960	698	707
11	999546	454	999514	486	377	345
12	999764	236	999792	208	160	188
13	999883	117	999865	135	105	87
14	999938	62	999945	55	40	47
15	999971	29	999964	36	31	24
16	999988	12	999991	9	8	11
17	999996	4	999991	9	8	3
18	999997	3	999999	1	1	3
19	999998	2	999997	3	3	2
20	1000000	0	999999	1	1	0
21	1000000	0	1000000	0	0	0
22	999999	1	1000000	0	0	1
23	1000000	0	1000000	0	0	0
24	1000000	0	1000000	0	0	0
25	1000000	0	1000000	0	0	0
26	1000000	0	1000000	0	0	0
27	1000000	0	1000000	0	0	0
28	1000000	0	1000000	0	0	0
29	1000000	0	1000000	0	0	0
30	1000000	0	1000000	0	0	0
31	1000000	0	1000000	0	0	0
32	1000000	0	1000000	0	0	0
33	1000000	0	1000000	0	0	0
34	1000000	0	1000000	0	0	0
35	1000000	0	1000000	0	0	0
36	1000000	0	1000000	0	0	0
37	1000000	0	1000000	0	0	0
38	1000000	0	1000000	0	0	0
39	1000000	0	1000000	0	0	0
40	1000000	0	1000000	0	0	0
41	1000000	0	1000000	0	0	0
42	1000000	0	1000000	0	0	0
43	1000000	0	1000000	0	0	0
44	1000000	0	1000000	0	0	0
45	1000000	0	1000000	0	0	0
46	1000000	0	1000000	0	0	0
47	1000000	0	1000000	0	0	0

S	A_infeasible	A_feasible	M_infeasible	M_feasible	A_made_feasible	A_made_infeasible
48	1000000	0	1000000	0	0	0
49	1000000	0	1000000	0	0	0
50	1000000	0	1000000	0	0	0

Hence, in general, σ^2_γ does not appear to affect feasibility in pure species interaction networks[8].

Stability given targeted manipulation of γ (genetic algorithm)

The figure below compares the stability of large complex systems given $\gamma = 1$ versus targeted manipulation of γ elements. For each S, 100000 complex systems are randomly generated. Stability of each complex system is tested given variation in γ using a genetic algorithm to maximise the effect of γ values on increasing stability, as compared to stability in an otherwise identical system in which γ is the same for all components. Blue bars show the number of stable systems in the absence of component response rate variation, while red bars show the number of stable systems that can be generated if component response rate is varied to maximise system stability. The black line shows the proportion of systems that are stable when component response rate is targeted to increase stability, but would not be stable if $\sigma^2_\gamma = 0$. The y-axis shows the ln number of systems that are stable across $S = \{1, 2, ..., 39, 40\}$ for $C = 1$, and the proportion of systems wherein a targeted search of γ values successfully resulted in system stability.

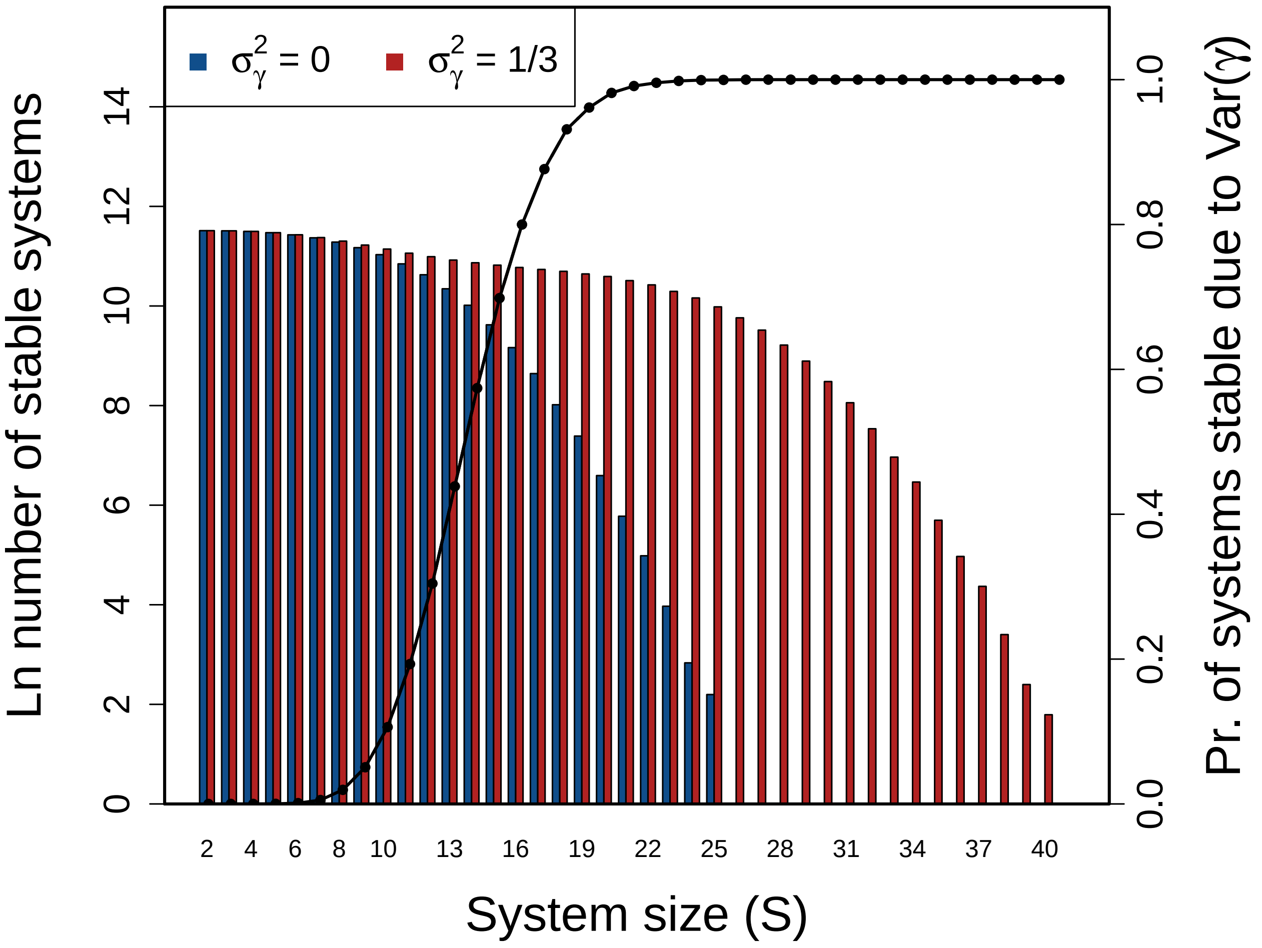

Stability results are also shown in the table below. Results for `A` indicate systems in which $\gamma = 1$, while `M` refers to systems in which the genetic algorithm searched for a set of γ values that stabilised the system.

S	A_unstable	A_stable	M_unstable	M_stable	A_stabilised	A_destabilised
2	26	99974	26	99974	0	0
3	358	99642	358	99642	0	0
4	1505	98495	1505	98495	0	0
5	3995	96005	3982	96018	13	0
6	8060	91940	7956	92044	104	0
7	13420	86580	12953	87047	468	1
8	20518	79482	18940	81060	1578	0
9	28939	71061	25148	74852	3793	2
10	38241	61759	30915	69085	7327	1
11	48682	51318	36398	63602	12286	2
12	58752	41248	40710	59290	18043	1
13	68888	31112	44600	55400	24289	1
14	77651	22349	47528	52472	30124	1
15	84912	15088	49971	50029	34942	1
16	90451	9549	52274	47726	38178	1
17	94332	5668	54124	45876	40209	1
18	96968	3032	55831	44169	41139	2
19	98384	1616	58079	41921	40305	0
20	99269	731	60181	39819	39088	0
21	99677	323	63338	36662	36339	0
22	99854	146	66350	33650	33504	0
23	99947	53	70478	29522	29469	0
24	99983	17	74121	25879	25862	0
25	99991	9	78364	21636	21627	0
26	99999	1	82635	17365	17364	0
27	100000	0	86433	13567	13567	0
28	100000	0	89951	10049	10049	0
29	100000	0	92716	7284	7284	0
30	100000	0	95171	4829	4829	0
31	100000	0	96844	3156	3156	0
32	100000	0	98128	1872	1872	0
33	100000	0	98941	1059	1059	0
34	100000	0	99358	642	642	0
35	100000	0	99702	298	298	0
36	100000	0	99856	144	144	0
37	100000	0	99921	79	79	0
38	100000	0	99970	30	30	0
39	100000	0	99989	11	11	0
40	100000	0	99994	6	6	0

The distributions of nine γ vectors from the highest S values are shown below. This comparison shows the high number of stable $\mathbf{M}$ that can be produced through a targeted search of γ values, and suggests that many otherwise unstable systems could potentially be stabilised by an informed manipulation of their component response times. Such a possibility might conceivably reduce the dimensionality of problems involving stability in social-ecological or economic systems.

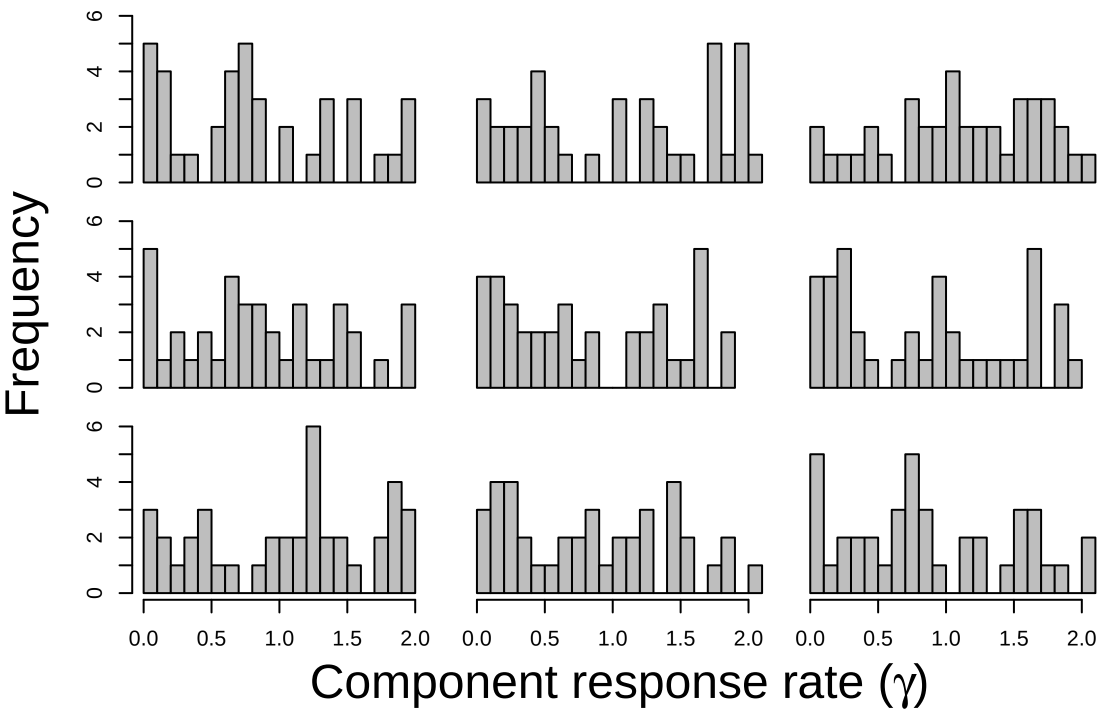

The distribution of γ values found by the genetic algorithm is uniform. A uniform distribution was used to initialise γ values, so there is therefore no evidence that a particular distribution of γ is likely to be found to stabilise a matrix $\mathbf{M}$.

Consistency with Gibbs et al. (2018)

The question that I address in the main text is distinct from that of Gibbs et al.[9], who focused instead on the effect of a diagonal matrix of biological species densities $\mathbf{X}$ on a community matrix $\mathbf{M}$ given a species interaction matrix $\mathbf{A}$. This is modelled as below,

$$\mathbf{M} = \mathbf{X}\mathbf{A}.$$

Mathematically, the above is identical to my model in the main text where the system $\mathbf{M}$ is defined by component interaction strengths $\mathbf{A}$ and individual component response rates $\boldsymbol{\gamma}$,

$$\mathbf{M} = \gamma\mathbf{A}.$$

I focused on the probability of observing a stable versus unstable system given variation in γ as system complexity ($\sigma\sqrt{SC}$) increased. I increased system complexity by holding C and σ constant and incrementally increasing S to obtain numerical results. In contrast, Gibbs et al.[9] applied analytical techniques to instead focus on a different question concerning the effect of γ on the stability of $\mathbf{M}$ given $\mathbf{A}$ as $S \to \infty$, with σ scaled so that $\sigma = 1/\sqrt{S}$. Under such scaling, Gibbs et al.[9] showed that the effect of γ on stability should decrease exponentially as S increases, which I demonstrate below by running simulations in which $\sigma = 1/\sqrt{S}$.

S	A_unstable	A_stable	M_unstable	M_stable	A_stabilised	A_destabilised
2	3111	96889	3111	96889	0	0
3	5203	94797	5237	94763	1	35
4	6743	93257	6818	93182	6	81

S	A_unstable	A_stable	M_unstable	M_stable	A_stabilised	A_destabilised
5	7889	92111	8005	91995	20	136
6	8834	91166	8991	91009	55	212
7	9885	90115	10072	89928	81	268
8	10516	89484	10764	89236	108	356
9	11135	88865	11383	88617	145	393
10	11819	88181	12095	87905	181	457
11	12414	87586	12700	87300	213	499
12	12865	87135	13136	86864	283	554
13	13530	86470	13836	86164	324	630
14	13745	86255	14042	85958	362	659
15	14401	85599	14720	85280	387	706
16	14793	85207	15123	84877	428	758
17	15004	84996	15356	84644	444	796
18	15361	84639	15735	84265	472	846
19	16062	83938	16303	83697	592	833
20	15814	84186	16184	83816	566	936
21	16171	83829	16492	83508	640	961
22	16671	83329	17049	82951	641	1019
23	17000	83000	17291	82709	718	1009
24	17411	82589	17666	82334	765	1020
25	17414	82586	17742	82258	783	1111
26	17697	82303	18027	81973	806	1136
27	18010	81990	18316	81684	880	1186
28	18584	81416	18735	81265	1008	1159
29	18401	81599	18572	81428	942	1113
30	18497	81503	18754	81246	952	1209
31	18744	81256	18942	81058	991	1189
32	18936	81064	19194	80806	1022	1280
33	19174	80826	19346	80654	1113	1285
34	19477	80523	19632	80368	1120	1275
35	19659	80341	19777	80223	1206	1324
36	19883	80117	19929	80071	1275	1321
37	20275	79725	20348	79652	1308	1381
38	20067	79933	20190	79810	1275	1398
39	20416	79584	20516	79484	1340	1440
40	20370	79630	20489	79511	1359	1478
41	20295	79705	20430	79570	1382	1517
42	20767	79233	20839	79161	1418	1490
43	20688	79312	20705	79295	1471	1488
44	21049	78951	21028	78972	1555	1534
45	21114	78886	21034	78966	1572	1492
46	21163	78837	21195	78805	1463	1495
47	21373	78627	21353	78647	1535	1515
48	21338	78662	21285	78715	1632	1579
49	21547	78453	21566	78434	1575	1594
50	21738	78262	21633	78367	1636	1531
51	21967	78033	21892	78108	1698	1623

Above table results can be compared to those of the main results. Note that 100000 (not 1 million), simulations are run to confirm consistency with Gibbs et al.[9]. The difference between my model and Gibbs et al.[9] is that in the latter, $\sigma\sqrt{SC} = 1$ remains constant with increasing S. In the former, $\sigma\sqrt{SC}$ increases with S, so the expected complexity of the system also increases accordingly. Consequently, for the scaled σ in the table

above, systems are not more likely to be stabilised by γ as S increases, consistent with Gibbs et al.[9]. Note that overall stability does decrease with increasing S due to the increased density of eigenvalues (see below).

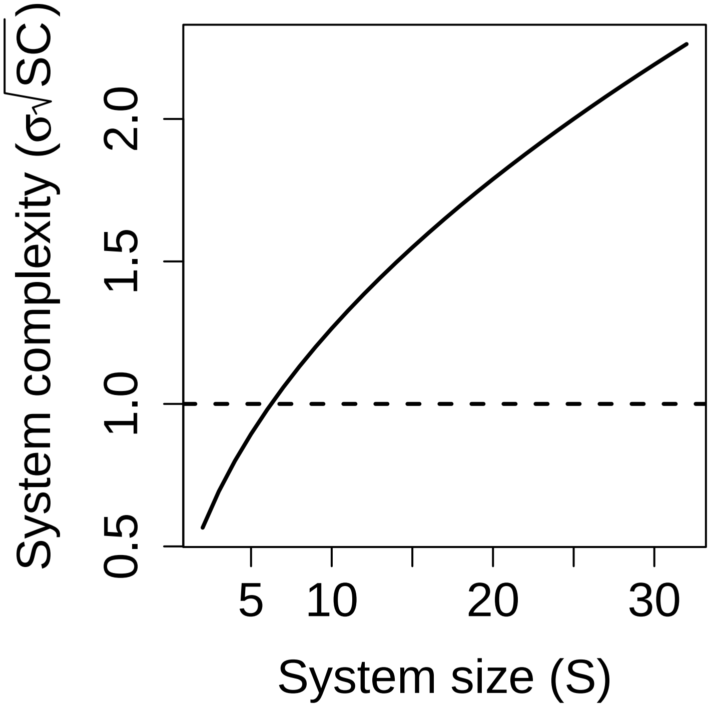

Complexity as a function of S in the main text (solid) versus in Gibbs et al.[9] (dashed).

When the complexity is scaled to $\sigma\sqrt{SC} = 1$, an increase in S increases the eigenvalue density within a circle with a unit radius centred at $(-1, 0)$ on the complex plane. As $S \to \infty$, this circle becomes increasingly saturated. Gibbs et al.[9] showed that a diagonal matrix γ will have an exponentially decreasing effect on stability with increasing S. Increasing S is visualised below, first with a system size $S = 100$.

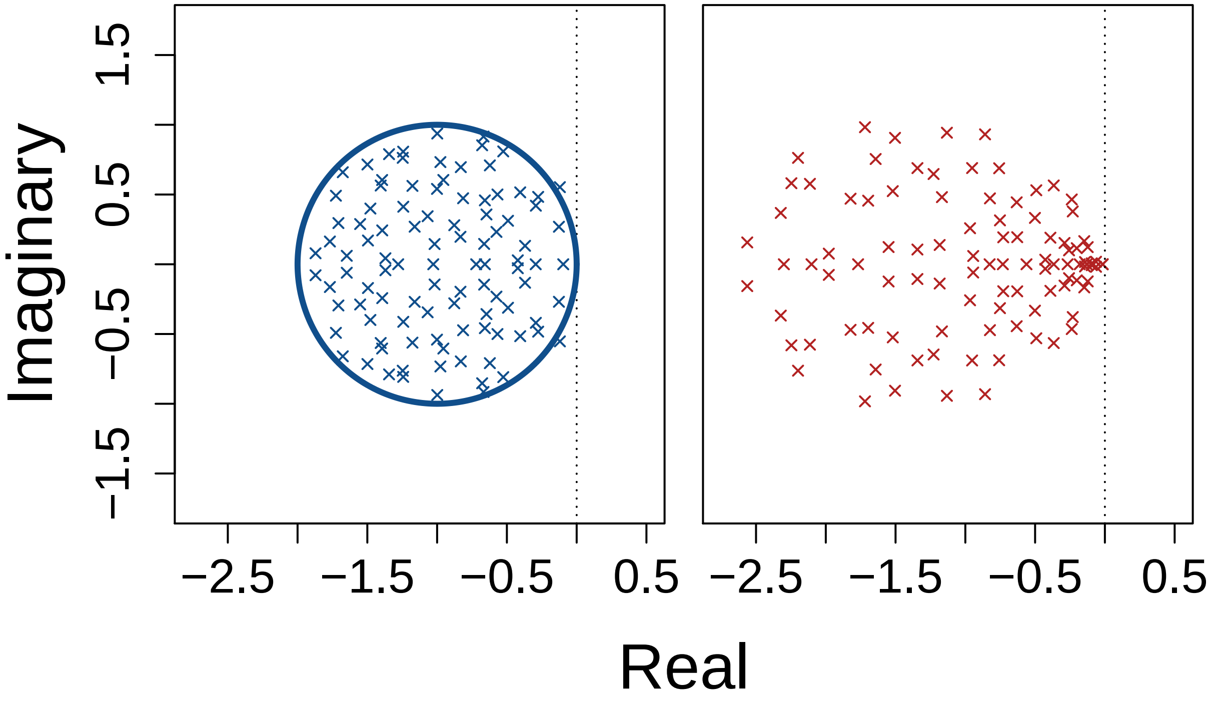

The left panel above shows the distribution of eigenvalues; the blue ellipse shows the unit radius within which eigenvalues are expected to be contained. The right panel shows how eigenvalue distributions change given $\gamma \sim \mathcal{U}(0, 2)$. The vertical dotted line shows the threshold of stability, $\Re = 0$. Increasing to $S = 200$, the scaling $\sigma = 1/\sqrt{S}$ maintains the expected distribution of eigenvalues but increases eigenvalue density.

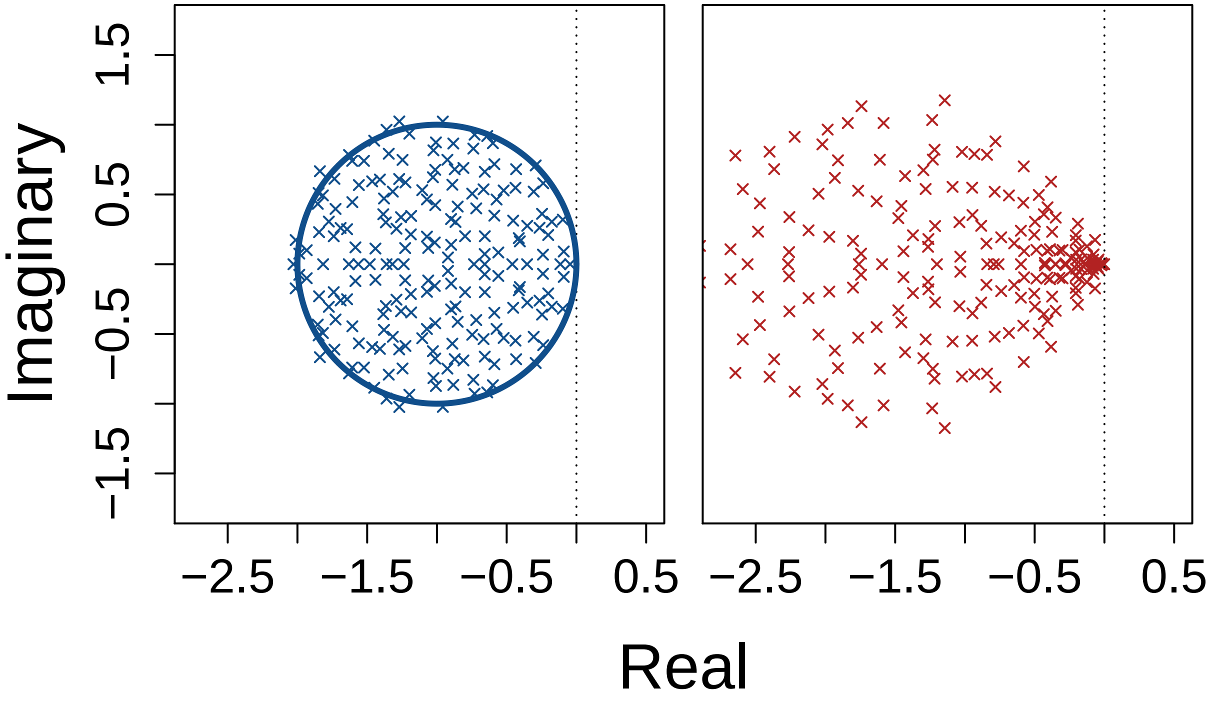

We can increase the system size to $S = 500$ and see the corresponding increase in eigenvalue density.

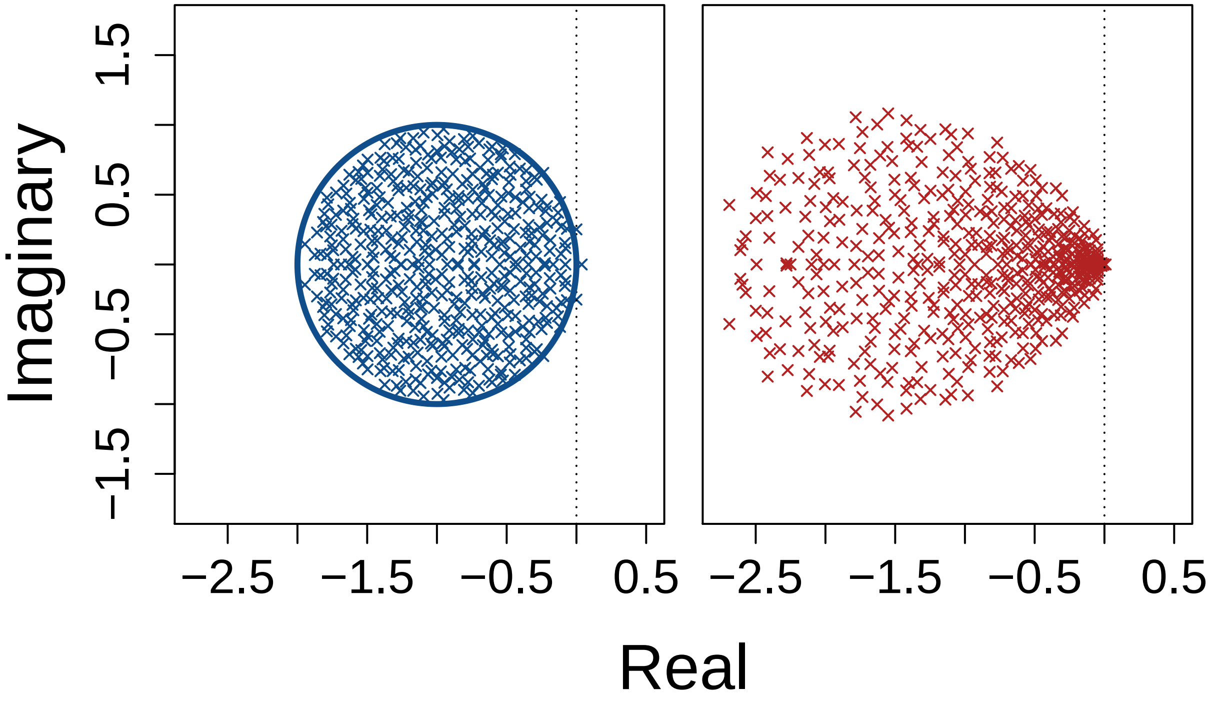

Finally, below shows a increase in system size to $S = 1000$.

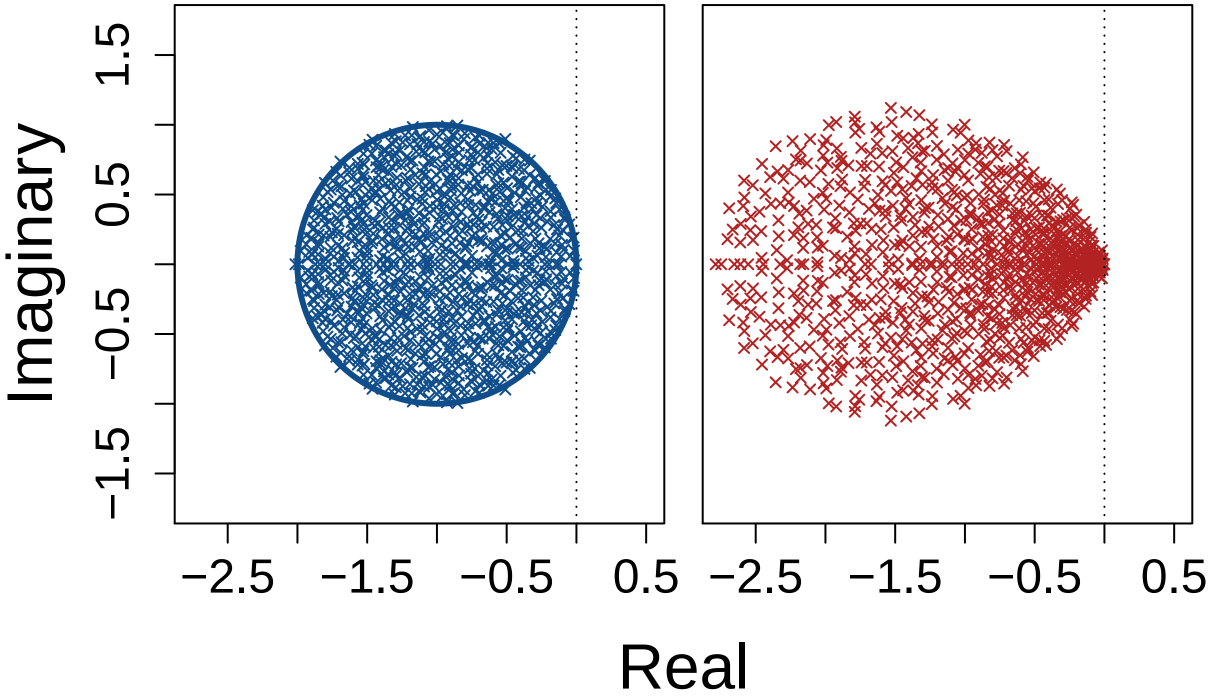

In contrast, in the model of the main text, the complexity of system is not scaled to $\sigma\sqrt{SC} = 1$. Rather, the density of eigenvalues within a circle centred at $(-1, 0)$ with a radius $\sigma\sqrt{SC}$ is held constant such that there are $S/\pi(\sigma\sqrt{SC})^2$ eigenvalues per unit area of the circle. As S increases, so does the expected complexity of the system, but the density of eigenvalues remains finite causing error around this expectation. Below shows a system where $S = 100$, $C = 0.0625$, and $\sigma = 0.4$, where $\sigma\sqrt{SC} = 1$ (identical to the first example distribution above in which $S = 100$ and $\sigma = 1/\sqrt{S}$).

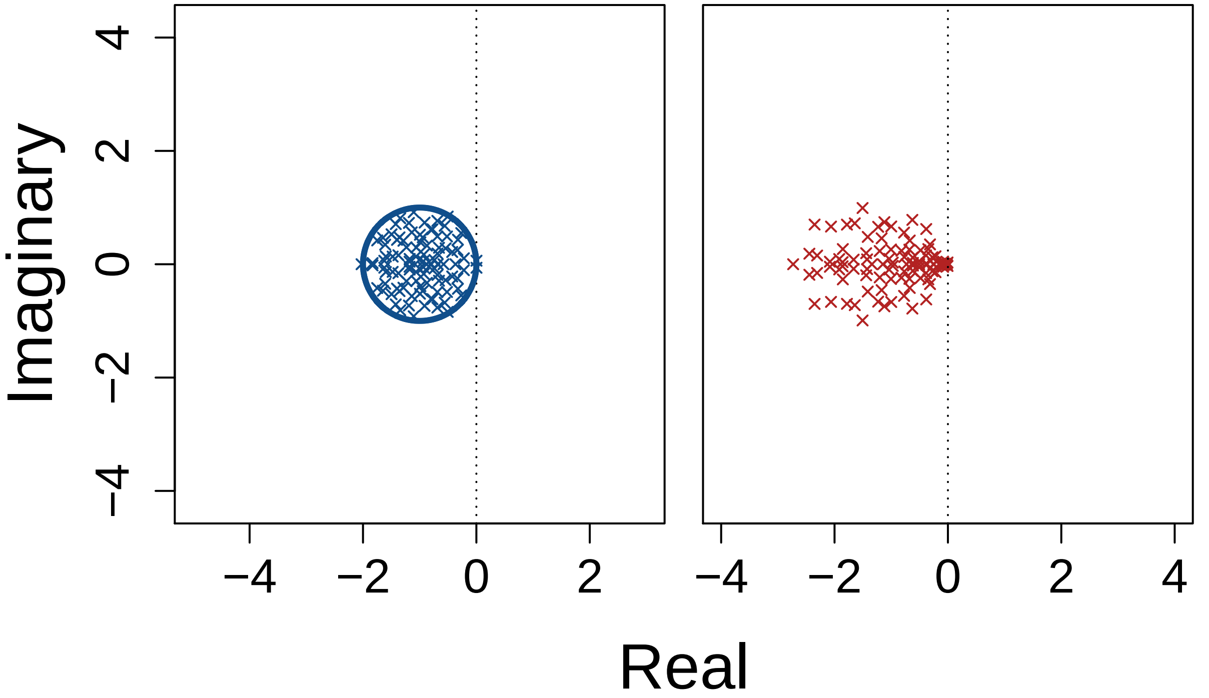

Now when S is increased to 200 while keeping $C = 0.0625$ and $\sigma = 0.4$, the area of the circle within which eigenvalues are contained increases to keep the density of eigenvalues constant.

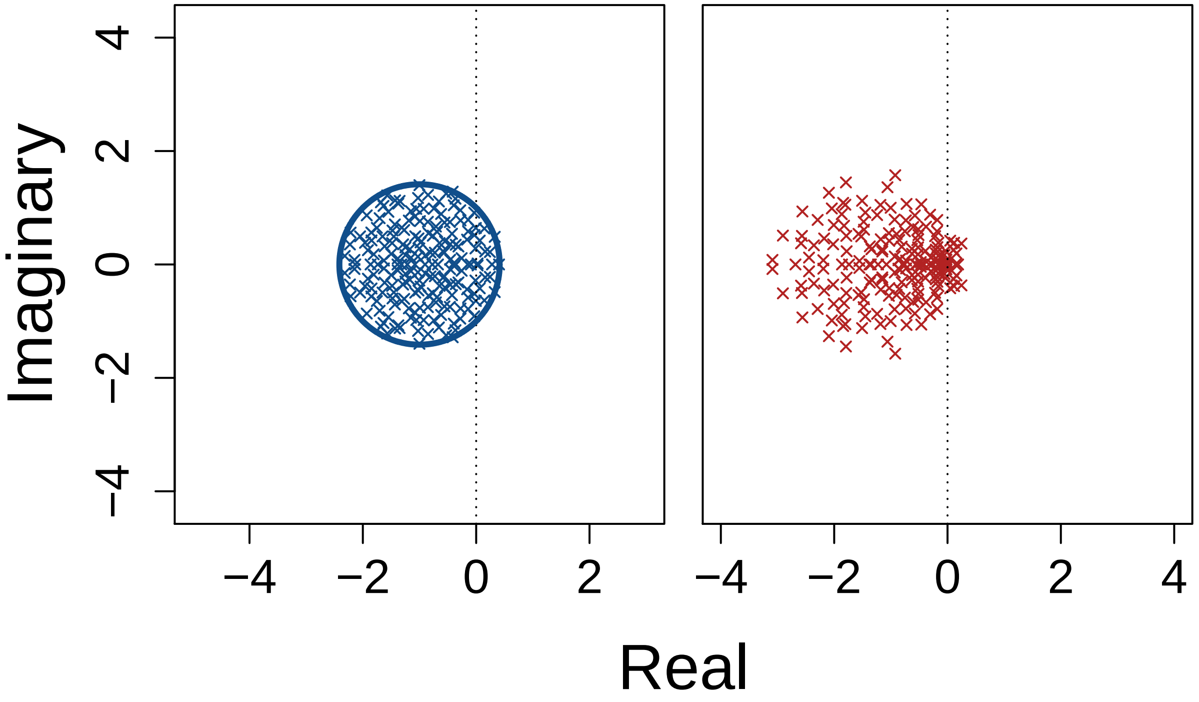

Note that the expected distribution of eigenvalues increases so that the threshold $\Re = 0$ is exceeded. Below, system size is increased to $S = 500$.

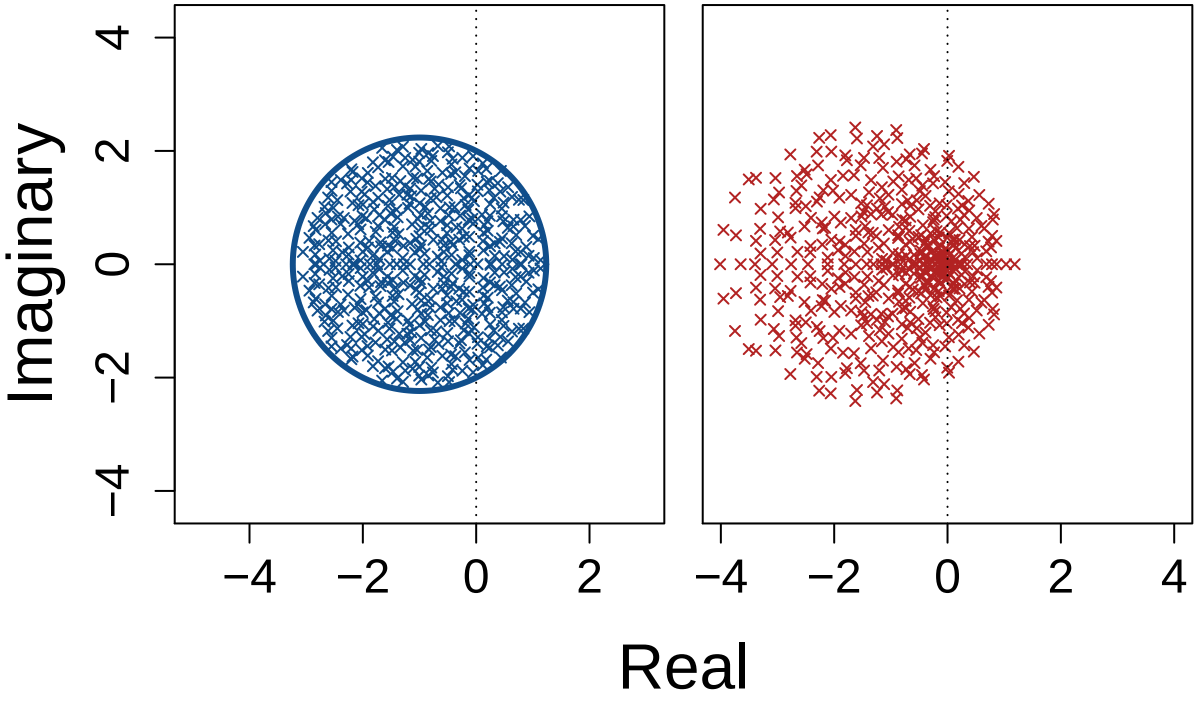

Finally, $S = 1000$ is shown below. Again, the density of eigenvalues per unit remains constant at ca 2, but the system has increased in complexity such that some real components of eigenvalues are almost assured to be greater than zero.

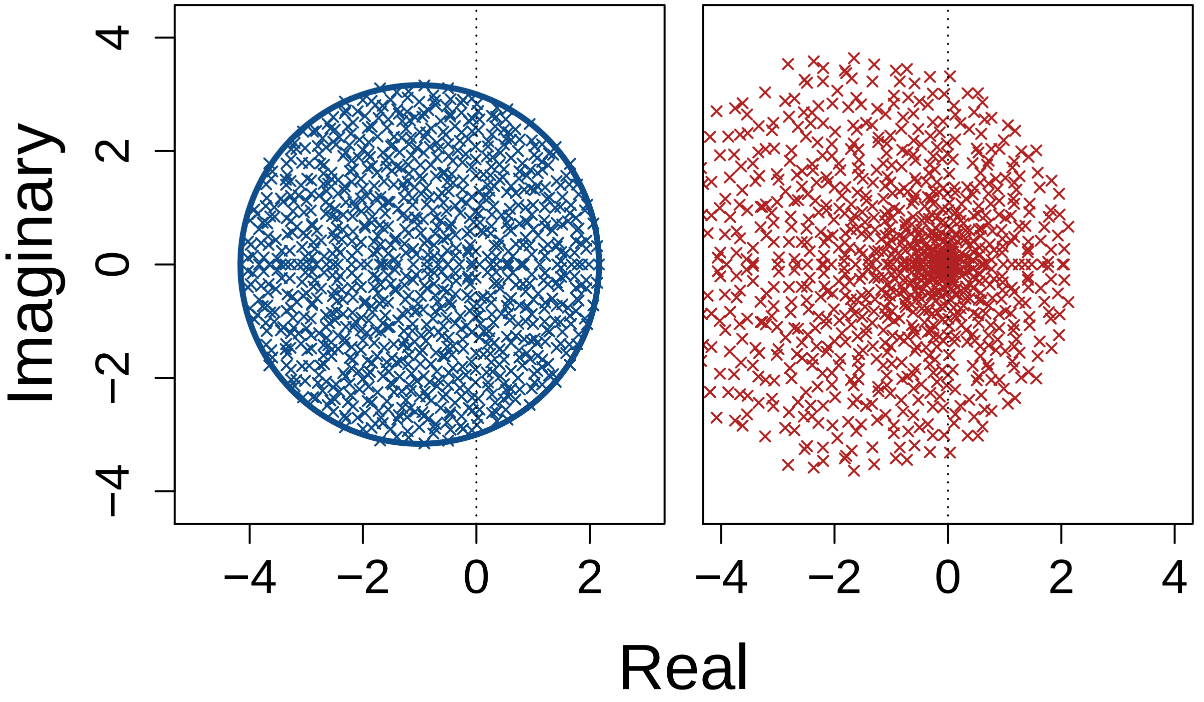

Reproducing simulation results

All results in the main text and the literature cited can be reproduced using the RandomMatrixStability R package, which can be downloaded as instructed at the beginning of this Supplemental Information document. The most relevant R functions for reproducing simulations include the following:

1. **`rand_gen_var`**: Simulates random complex systems and cascade food webs
2. **`rand_rho_var`**: Simulates random complex systems across a fixed correlation of $\rho = cor(A_{ij}, A_{ji})$
3. **`rand_gen_swn`**: Simulates randomly generated small-world networks
4. **`rand_gen_sfn`**: Simulates randomly generated scale-free networks
5. `Evo_rand_gen_var`: Use a genetic algorithm to find stable random complex systems

For the functions 1-4 above, R output will be a table of results. Below describes the headers of this table to more clearly explain what is being reported.

Header	Description	Header_cont.	Description_cont.
S	The system size	A_rho	Corr. between elements A[ij] and A[ji]
A_unstable	No. of A that were unstable	M_rho	Corr. between elements M[ij] and M[ji]
A_stable	No. of A that were stable	rho_diff	Diff. between A and M rho values
M_unstable	No. of M that were unstable	rho_abs	Diff. between A and M rho magnitudes
M_stable	No. of M that were stable	complex_A	Complexity of A
A_stabilised	No. of A stabilised by gamma	complex_M	Complexity of M
A_destabilised	No. of A destabilised by gamma	A_eig	Expected real part of leading A eigenvalue
A_infeasible	No. of A that were infeasible	M_eig	Expected real part of leading M eigenvalue
A_feasible	No. of A that were feasible	LR_A	Lowest obs. real part of leading A eigenvalue
M_infeasible	No. of M that were infeasible	UR_A	Highest obs. real part of leading A eigenvalue
M_feasible	No. of M that were feasible	LR_M	Lowest obs. real part of leading M eigenvalue
A_made_feasible	No. of A made feasible by gamma	UR_M	Highest obs. real part of leading M eigenvalue
A_made_infeasible	No. of A made infeasible by gamma	C	Obs. network connectance

Note that output from `Evo_rand_gen_var` only includes the first seven rows of the table above, and **`rand_gen_var`** does not include C (which can be defined as an argument). All results presented here and in

the main text are available in the inst/extdata folder of the RandomMatrixStability R package.

Literature cited

1. May, R. M. Will a large complex system be stable? *Nature* **238,** 413–414 (1972).

2. Allesina, S. & Tang, S. Stability criteria for complex ecosystems. *Nature* **483,** 205–208 (2012).

3. Allesina, S. *et al.* Predicting the stability of large structured food webs. *Nature Communications* **6,** 7842 (2015).

4. Watts, D. J. & Strogatz, S. H. Collective dynamics of 'small world' networks. *Nature* **393,** 440–442 (1998).

5. Albert, R. & Barabási, A. L. Statistical mechanics of complex networks. *Reviews of Modern Physics* **74,** 47–97 (2002).

6. Solow, A. R. & Beet, A. R. On lumping species in food webs. *Ecology* **79,** 2013–2018 (1998).

7. Williams, R. J. & Martinez, N. D. Simple rules yield complex food webs. *Nature* **404,** 180–183 (2000).

8. Serván, C. A., Capitán, J. A., Grilli, J., Morrison, K. E. & Allesina, S. Coexistence of many species in random ecosystems. *Nature Ecology and Evolution* **2,** 1237–1242 (2018).

9. Gibbs, T., Grilli, J., Rogers, T. & Allesina, S. The effect of population abundances on the stability of large random ecosystems. *Physical Review E - Statistical, Nonlinear, and Soft Matter Physics* **98,** 022410 (2018).
